# Unraveling the Role of Acetic Acid Bacteria Comparing Two Acetification Profiles From Natural Raw Materials: A Quantitative Approach in *Komagataeibacter europaeus*

**DOI:** 10.3389/fmicb.2022.840119

**Published:** 2022-04-29

**Authors:** Juan J. Román-Camacho, Juan C. Mauricio, Inés M. Santos-Dueñas, Teresa García-Martínez, Isidoro García-García

**Affiliations:** ^1^Department of Agricultural Chemistry, Edaphology and Microbiology, University of Córdoba, Córdoba, Spain; ^2^Department of Inorganic Chemistry and Chemical Engineering, Institute of Nanochemistry (IUNAN), University of Córdoba, Córdoba, Spain

**Keywords:** *Komagataeibacter europaeus*, vinegar, fine wine, craft beer, proteomics, submerged culture

## Abstract

The industrial production of vinegar is carried out by the activity of a complex microbiota of acetic acid bacteria (AAB) working, mainly, within bioreactors providing a quite specific and hard environment. The “omics” sciences can facilitate the identification and characterization analyses of these microbial communities, most of which are difficult to cultivate by traditional methods, outside their natural medium. In this work, two acetification profiles coming from the same AAB starter culture but using two natural raw materials of different alcoholic origins (fine wine and craft beer), were characterized and compared and the emphasis of this study is the effect of these raw materials. For this purpose, the composition and natural behavior of the microbiota present throughout these profiles were analyzed by metaproteomics focusing, mainly, on the quantitative protein profile of *Komagataeibacter europaeus*. This species provided a protein fraction significantly higher (73.5%) than the others. A submerged culture system and semi-continuous operating mode were employed for the acetification profiles and liquid chromatography with tandem mass spectrometry (LC-MS/MS) for the protein analyses. The results showed that neither of two raw materials barely modified the microbiota composition of the profiles, however, they had an effect on the protein expression changes in different biological process. A molecular strategy in which *K. europaeus* would prevail over other species by taking advantage of the different features offered by each raw material has been suggested. First, by assimilating the excess of inner acetic acid through the TCA cycle and supplying biosynthetic precursors to replenish the cellular material losses; second, by a previous assimilation of the excess of available glucose, mainly in the beer medium, through the glycolysis and the pentose phosphate pathway (PPP); and third, by triggering membrane mechanisms dependent on proton motive force to detoxify the cell at the final moments of acetification. This study could complement the current knowledge of these bacteria as well as to expand the use of diverse raw materials and optimize operating conditions to obtain quality vinegars.

**Clinical Trial Registration:**

[www.ClinicalTrials.gov], identifier [PXD031147].

## Introduction

The industrial elaboration of vinegar is carried out through an acetification process from an alcoholic raw material obtaining a final product with high acetic acid content. The incomplete oxidation of the ethanol into acetic acid is performed by acetic acid bacteria (AAB), strictly aerobic microorganisms that, among their several biotechnological applications, are primarily responsible for this process of biotransformation that occurs within industrial reactors (García-García et al., 2007; Mamlouk and Gullo, 2013).

The quality of the vinegar depends on many factors including the microbial composition, the raw material, and operating conditions (Mas et al., 2014; Li et al., 2015). Regarding microbial composition, several studies have demonstrated that vinegar is a product resulting from the metabolism of a complex AAB microbiota, not by pure species (Trček et al., 2016; Román-Camacho et al., 2020). This microbiota is mostly composed of species from the genera *Acetobacter* and *Komagataeibacter* (many of them relocated from *Gluconacetobacter*) which are imposed because of their high capabilities for vinegar production, although species from other genera might coexist with the best-adapted ones (Gullo et al., 2014; Wang et al., 2015). The raw material employed as acetification substrate plays an essential role in the quality of the final product. High-quality wines allow to elaborate some of the most appreciated vinegars in the world, however, other alcoholic substrates including cereals (rice, malt, wheat, corn, and among others), fruits, and apple cider are also well-known (Hidalgo et al., 2013; Trček et al., 2016; Zhang et al., 2019; Kandylis et al., 2021; Peng et al., 2021). Conversely, vinegar is mainly produced at the industrial scale by submerged cultures in reactors that continuously supply very fine air bubbles into the medium as an aeration mechanism. The submerged system has several advantages over other techniques, such as solid-state fermentation or surface fermentation including high yield and process speed (Gullo et al., 2014). Through a semi-continuous operating mode, in which each cycle starts by loading the tank with fresh medium to a preset volume and finishes when a part of the volume is unloaded after depleting ethanol to an also preset concentration, high productivity and stability are ensured (Jiménez-Hornero et al., 2020). This working mode allows part of the biomass produced in each cycle to rapidly start the next one. Also, the operational variables can be used to maintain the average substrate and product concentrations within appropriate ranges for AAB to operate, which in turn, facilitates self-selection and adjustment to the specific medium (García-García et al., 2019).

The particular growing conditions and metabolic characteristics of AAB hinder their isolation outside the environments in which they carry out their activity fully (Fernández-Pérez et al., 2010; Mamlouk and Gullo, 2013). This fact limits the study of the richness and biodiversity of these microbiota that inhabit aggressive media as is the case of vinegar. The “omics” sciences can facilitate the analysis of the identification and function of complex microbiomes and resolve the hurdles of traditional methods for the characterization of either non-cultivable or hard to cultivate microorganisms (Andrés-Barrao et al., 2016; Xia et al., 2016; Zhu et al., 2018; Jiang et al., 2019; Verce et al., 2019). Recently, the microbiota of an acetification process using an alcohol medium as a reference has been characterized at a metaproteomic level (Román-Camacho et al., 2020, 2021). The *Komagataeibacter* species were predominant throughout the process and *K. europaeus* provided the major fraction of proteins, far above the others. This species has been described as one of the most suitable AAB for the industrial production of vinegar because of its growing conditions that include high ethanol-oxidizing ability, acetic acid requirement, and tolerance to both low [7–9% (w/v)] and high acidity levels [10–20% (w/v)] (Trček et al., 2007; Yamada et al., 2012; Gullo et al., 2014; Peng et al., 2021).

The present work aims to characterize and compare two acetification profiles using the same starter culture, coming from an acetification of previous works (Román-Camacho et al., 2020, 2021) making alcohol vinegar, but using different raw materials. For this purpose, the composition and natural behavior of the microbiota present throughout both processes were compared employing a metaproteomic analysis and especially, focusing on the protein profile of *K. europaeus* from an exhaustive quantitative approach. This species has been selected, as in our previous studies, because it provides a considerable amount (73.5%) of the metaproteome and plays an essential role in the microbial community function. A comparison of vinegar profiles using two natural raw materials (fine wine and craft beer), with a higher nutritional richness than the reference synthetic alcohol medium (Román-Camacho et al., 2020, 2021), under a strategy that employs a submerged culture and a semi-continuous operating mode, could elucidate the effect of the raw materials on the organoleptic properties and quality of industrially elaborated vinegars.

## Materials and Methods

### Raw Material

Two different alcoholic substrates were used as fermentation media: a dry fine wine from the Montilla-Moriles region (Bodegas Alvear S.A., Montilla, Córdoba, Spain) and a craft beer (Mahou-San Miguel, Córdoba, Spain). The dry fine wine contained an initial ethanol concentration of 15% (v/v) and an amino acids content of 0.72 ± 0.20 mM for L-proline, 0.24 ± 0.03 mM for L-aspartic acid, 0.23 ± 0.21 mM for ammonium ion, 0.21 ± 0.00 mM for L-γ-aminobutyric acid, 0.19 ± 0.02 mM for L-glutamic acid, 0.16 ± 0.01 mM for L-lysine, 0.15 ± 0.01 mM for L-arginine, 0.11 ± 0.01 mM for L-tyrosine, 0.06 ± 0.01 mM for L-leucine, 0.05 ± 0.01 mM for L-valine, 0.04 ± 0.01 mM for L-histamine, 0.03 ± 0.01 mM for L-glycine, 0.02 ± 0.01 mM for L-threonine, and 0.01 ± 0.01 mM for L-tryptophan. Conversely, the craft beer was obtained from a medium containing 35% of total sugars, remaining without fermenting 7% and composed, roughly, half, and half between maple syrup and muscovado sugar. The ethanol content was 17% (v/v) and the amino acids content of 3.66 ± 0.05 mM for L-γ-aminobutyric acid, 1.44 ± 0.03 mM for L-aspartic acid, 1.21 ± 0.02 mM for L-glutamic acid, 1.05 ± 0.02 mM for L-arginine, 0.92 ± 0.02 mM for ammonium ion, 0.90 ± 0.13 mM for L-proline, 0.55 ± 0.01 mM for L-glutamine, 0.47 ± 0.01 mM for L-glycine, 0.39 ± 0.01 mM for L-phenylalanine, 0.30 ± 0.01 mM for L-tryptophan, 0.18 ± 0.01 mM for L-leucine, 0.17 ± 0.01 mM for L-tyrosine, 0.13 ± 0.01 mM for L-histidine, 0.11 ± 0.03 mM for L-threonine, 0.04 ± 0.01 mM for L-histamine, and 0.02 ± 0.01 mM for L-lysine. Both raw materials were diluted with distilled water to adjust the ethanol concentration to the working conditions [≈ 10% (v/v)] reaching 9.8 ± 0.3 and 9.5 ± 0.3% (v/v) for fine wine and beer, respectively; the initial acetic acid concentration was of 0.2 ± 0.1% (w/v).

### Microorganism

The starter culture consisted of a mixed broth coming from a fully active acetification process making alcohol vinegar, concretely harvested from the end of the ethanol exhausting phase, see microbial composition in Supplementary File 1 (Román-Camacho et al., 2020). This original alcohol medium was composed of 10% ethanol, glucose (1 g/L), calcium pantothenate (13 mg/L), calcium citrate (0.1 g/L), potassium citrate (0.1 g/L), diammonium phosphate (0.5 g/L), magnesium sulfate (0.1 g/L), manganese sulfate (5 mg/L), and iron chloride (1 mg/L) following the method of Llaguno (1991) with yeast extract (0.25 g/L) and peptone (0.5 g/L) additionally supplied. A previous stage using each specific raw material, including several cycles of acetification, is necessary to adapt the inoculum and achieve a repetitive system behavior.

### Operating Mode

**FIGURE 1:**
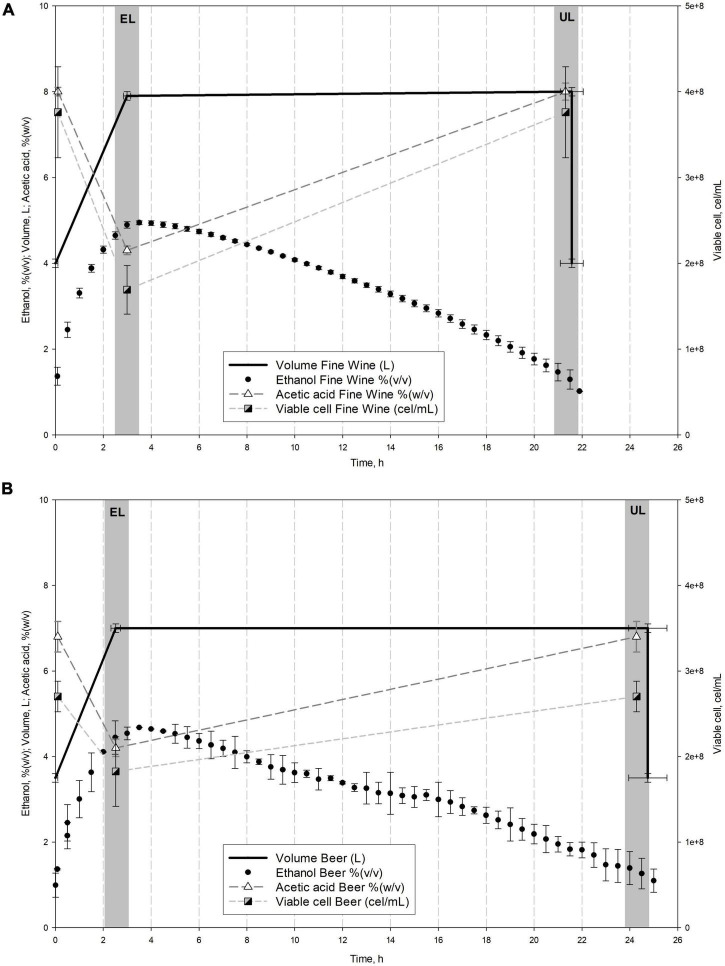
Comparison of the main system variables throughout fine wine **(A)** and beer **(B)** vinegar profiles. Mean values and standard deviation (*SD*) of the variables of stable cycles performed are represented. Sampling times (EL, end of loading; UL, just before unloading) are also shown.

Acetification cycles were carried out in a fully automated 8 L Frings bioreactor (Heinrich Frings GmbH & Co., KG, Bonn, Germany) working in a semi-continuous operating mode. Each cycle is started by a loading phase that replenishes the reactor with a fresh medium to the working volume (8 L) without exceeding a preset ethanol concentration of 5% (v/v). Then, an exhausting stage occurs depleting ethanol in the culture broth to a preset concentration of 1.0–1.5% (v/v). Finally, 50% of the volume is fast unloaded and the remaining content is used as inoculum of the next cycle. A constant temperature of 31^°^C, a fast-loading rate of 1.3 L/h, and an air-flow rate of 7.5 L/(h L medium) were employed. Sigmaplot 12.0 (Systat Software Inc., CA, United States) was used for graphical representation of the acetification profiles after monitoring the system data by LabView application (National Instruments, TX, United States). Figure 1 shows the profiles of the main variables.

### Sampling

Sampling was performed at two relevant times of the acetification cycle: at the end of the loading phase (EL), when final working volume or preset ethanol concentration of 5% (v/v) is reached, whichever occurs first; and just before the unloading phase, at the end of the ethanol exhaustion (UL). A total of 15 acetification cycles for each vinegar profile were performed including some previous cycles (7–10) necessary to achieve a semi-continuous repetitive state of the system. Six samples were harvested from fine wine vinegar: three at EL (cycles 12, 14, and 15) and three at UL (cycles 11, 13, and 14); and seven samples from beer vinegar: four at EL (cycles 11, 12, 13, and 14) and three at UL (cycles 12, 13, and 14).

### Analytical Methods

System variables including the volume of the medium (L), ethanol concentration % (v/v), and temperature (^°^C) were constantly measured using an EJA 110 differential pressure probe (Yokogawa Electric Corporation, Tokyo, Japan), an Alkosens ^®^ probe (Heinrich Frings GmbH & Co., KG, Bonn, Germany), and a temperature probe, respectively. The automatization of the system allows the continuous recording of data as well as testing the high reproducibility of the method. Acetic acid concentration %, (w/v) was determined by acid-base titration with 0.5 N NaOH. Viable cells concentration, the difference between total and no viable cells, were directly counted using a light microscope (Olympus BX51), a Neubauer chamber (Blaubrand™, 7178-10) with 0.02 mm depth and rhodium-coated bottom, and propidium iodide (VWR, Inc., PA, United States). Though the chamber was subdivided into 25 square groups, composed of 16 squares each, 5 square groups (0.04 mm^2^ each) on the diagonal were used for cell counting following the method of Baena-Ruano et al. (2006); samples were quantified by triplicate and standard error was calculated. These variables were exclusively measured at sampling times. The efficiency of the process was evaluated by mean acetification rate (*r*_*A*_) and global production of acetic acid (*p*_*A*_) which were calculated as follows:


$$r_{A}=\frac{\begin{array}{c} Finalaceticacidconcentration\left(\%,w/v\right)\\ \times U\,n\,l\,o\,a\,d\,e\,d\,v\,o\,l\,u\,m\,e\,\left(L\right) \end{array}}{\begin{array}{c} T\,o\,t\,a\,l\,c\,y\,c\,l\,e\,t\,i\,m\,e\,\left(h\right)\times M\,e\,a\,n\,c\,y\,c\,l\,e\,v\,o\,l\,u\,m\,e\,\left(L\right) \end{array}}$$

$$p_{A}=\frac{\begin{array}{c} Finalaceticacidconcentration\left(\%,w/v\right)\\ \times U\,n\,l\,o\,a\,d\,e\,d\,v\,o\,l\,u\,m\,e\,\left(L\right) \end{array}}{\begin{array}{c} T\,o\,t\,a\,l\,c\,y\,c\,l\,e\,t\,i\,m\,e\,\left(h\right) \end{array}}$$

### Proteomics

#### Sample Processing

Vinegar samples were harvested by directly unloading a volume of 300 mL from the pilot acetator, dividing it into six fractions of 50 mL each, and putting them in centrifuge tubes on ice. Cells were separated by centrifugation and then, twice cleaned using cold sterile distilled water; the resulting pellets were stored at −80^°^C. Then, cell extracts were broken by several cycles using glass beads and sonication after adding extraction buffer (100 mM Tris-HCl buffer pH 8.0, 2 mM dithiothreitol (DTT), 1 mM ethylenediaminetetraacetic acid (EDTA), and 1 mM phenylmethylsulphonyl fluoride (PMSF) supplemented with Protease Inhibitor Cocktail tablets). The protein fraction was precipitated, vacuum dried, solubilized, and its concentration was quantified by Bradford (1976) assays. A volume of each protein sample containing 50 μg was injected into LC-MS/MS analysis at Research Support Central Service (SCAI), University of Córdoba, Spain. All proteomic procedures were performed following the methodology previously developed by our group (Román-Camacho et al., 2020).

#### Protein Identification by Database Searching

Mass spectrometry raw data were processed using Proteome Discoverer (version 2.1.0.81, Thermo Fisher Scientific, MA, United States). MS/MS spectra were searched with SEQUEST engine against Uniprot.^1^ Peptides obtained from tryptic digestion were searched setting the following parameters: up to one missed cleavage, cysteine carbamidomethylation as a fixed modification, and methionine oxidation as a variable one. Precursor mass tolerance was 10 ppm while ion products were searched at 0.1 Da tolerances. Peptide spectral matches (PSM) validation was performed at a 1% FDR using a percolator based on *q*-values. Peptide quantification was carried out by calculating precursor ion areas by Precursor Ion Area Detector and normalizing by Total Peptide Amount mode of Proteome Discoverer. The parsimony law was applied to obtain protein groups and filtered to 1% FDR.

The mass spectrometry proteomics data have been deposited to the ProteomeXchange Consortium via the PRIDE [1] partner repository with the dataset identifier PXD031147.

#### Raw Data Analysis

Proteins identified in the metaproteome were first screened removing those with score < 2 and number of peptides ≤ 2. Then, proteins in at least 50% out of total samples (three or four) in at least one sampling time were maintained. Exclusive proteins were obtained by the difference between those aforementioned and those identified in at least 50% out of total samples in each sampling time. From those, a GO Term analysis using Uniprot and Gene Ontology (GO) annotation tool^2^ was performed to detail the metaproteome function. Subsequently, an enrichment analysis LC-MS^2^ of the proteome of *K. europaeus* was performed and quantitative changes throughout each acetification profile were compared. Protein values were normalized by dividing each one by the sample global intensity and then multiplied by the mean value of global intensity from all samples. First, those proteins obtained in at least 50% of samples in one sampling time were retained and plotted in an intersection diagram (“UpSetR” R library). For the hierarchical clustering and heat map analysis, proteins identified in at least 50% of samples in each sampling time were used. Mean quantification values were previously scaled, centered by z-score transformation, and then, Pearson correlation was applied with method “complete” (“hclust” function in stats package from R). One-Way ANOVA followed by HSD Tukey’s test was calculated by R functions “lm” and “anova” and *q*-value was used to calculate *p*-value multiple testing correction. Proteins identified only in one biological replicate were eliminated from the overall count.

Furthermore, the biological function of the protein clusters was studied by building protein-protein interaction network maps (INM) by using STRING database v11.^3^ High confidence interaction (score = 0.70--0.90) and protein annotations based on the databases Uniprot (see text footnote 1) and KEGG^4^ were used (see Supplementary File 2). Because *K. europaeus* is not available in the database, as in previous works (Román-Camacho et al., 2021), *K. xylinus* E25, a closely related species (MUM index of 0.21, according to Ryngajłło et al., 2018), was used as a model organism due to the high genome homology.

## Results

### Description of Fine Wine and Beer Acetification Profiles: A Comparison

Figure 1 shows a comparison of the mean cycle of main system variables throughout fine wine (Figure 1A) and beer (Figure 1B) profiles, while Table 1 lists the mean values of the aforementioned variables. The fine wine profile showed a fast-loading phase up to reach the working volume (8.0 ± 0.1 L) and an ethanol concentration of 4.9 ± 0.0% (v/v) at the end of the stage, at 3.0 ± 0.0 h. An exhausting phase started with the depletion of ethanol content up to 1.3 ± 0.3% (v/v) and just then, 50% of the reactor volume was unloaded (4.0 ± 0.1 L), at 21.4 ± 0.1 h. During this period, both acetic acid concentration [from 4.3 ± 0.0 to 7.9 ± 0.2% (w/v)] and cell viable concentration (from 1.43 ± 0.33 to 1.47 ± 0.28 × 10^8^ cel/mL) were increased. The beer profile was operated with a final working volume of 7.0 ± 0.2 L because of the excessive foaming. First, a continuous fast loading was performed to the aforementioned volume and an ethanol concentration of 4.7 ± 0.2% (v/v), both achieved at 2.8 ± 0.4 h. The second phase (24.3 ± 1.1 h) concluded when ethanol concentration was depleted to 1.2 ± 0.1% (v/v) and 50% of the volume of the medium was unloaded (3.5 ± 0.1 L). At the same time, the acetic acid concentration [from 4.2 ± 0.4 to 6.8 ± 0.7% (w/v)] and cell viability (from 0.84 ± 0.70 to 1.05 ± 0.70 × 10^8^ cel/mL) were increased throughout this exhausting period. The efficiency of each acetification profile was evaluated by the mean acetification rate (*r*_*A*_) and global production of acetic acid (*p*_*A*_), both calculated as described in section “Analytical Methods” (see Table 1).

**TABLE 1:** Main variables of the acetification profile including both the system variables constantly monitored and those exclusively measured at sampling times.

	Variable	FW_EL	FW_UL	B_EL	B_UL
Mean ± *SD*	Cycle time (h)	3.0 ± 0.0	21.4 ± 0.1	2.8 ± 0.4	24.3 ± 1.1
	Volume (L)	8.0 ± 0.1	8.0 ± 0.1	7.0 ± 0.2	7.0 ± 0.2
	Ethanol (% v/v)	4.9 ± 0.0	1.3 ± 0.3	4.7 ± 0.2	1.2 ± 0.1
	Acetic acid (% w/v)	4.3 ± 0.0	7.9 ± 0.2	4.2 ± 0.4	6.8 ± 0.7
	Viable cell (10^8^ cel/mL)	1.43 ± 0.33	1.47 ± 0.28	0.84 ± 0.70	1.05 ± 0.70

		**FW**		**B**	

	Mean acetification rate (*r*_*A*_) [g acetic acid/(L h)]	0.19 ± 0.01		0.16 ± 0.01	
	Global acetic acid production (*p*_*A*_) (g acetic acid/h)	15.2 ± 0.5		11.3 ± 0.5	

*Data show mean values of all variables at the sampling times (FW_EL, FW_UL, B_EL, B_UL) and their standard deviation (SD). Variables used to obtain the acetification efficiency of each profile are included.*

It is interesting to note that the two raw materials used show some significant differences to evaluate the behavior of the microbiota as a function of the available nutrients. In particular, the significant presence of sugars in the craft beer medium could lead, as it will be discussed later in this work, to the activation of several metabolic pathways aimed at taking advantage of this resource. Similarly, fine wine, a substrate whose suitability as an acetification medium is well known, not only allows higher acetification rates, but also higher final acidity values, which leads to harsher environmental conditions. This fact can trigger the activation of metabolic pathways other than those mentioned above in response to stress.

### Comparison of the Metaproteome of Fine Wine and Beer Vinegar

#### Microbial Composition

A total of 1,069 (EL, 934; UL, 945) and 1,268 (EL, 1,226; UL, 1,110) proteins were identified in the LC/MS-MS analysis in fine wine and beer vinegar samples, respectively, after removing contaminants and those proteins not found in at least 50% of the samples in at least one sampling time (see Supplementary File 1). Although proteins belonging to 84 different species from the *Acetobacteraceae* family were identified, only 13 of them constituted around 90% of the metaproteome (see Supplementary Table 1): 11 species from the *Komagataeibacter* genus (*K. europaeus*, *K. xylinus*, *K. intermedius*, *K. rhaeticus*, *K. diospyri*, *K. swingsii*, *K. medellinensis*, *K. nataicola*, *K. oboediens*, *K.* sp., and *K. sucrofermentans*) as well as *Acetobacter* sp. and *Gluconacetobacter* sp.; *K. europaeus* was the most abundant species providing the largest amount of proteins (73.5%: FW_EL, 75.9%; FW_UL, 75.0%; B_EL, 70.6%; B_UL, 72.6%), far above the rest of species. No relevant differences regarding the composition of the microbiota were observed between sampling times and profiles. Since none of the remaining species exceeds a mean frequency of 0.5%, the functional metaproteome analysis is mainly focused on this major amount, which is considered sufficiently representative of the total (Román-Camacho et al., 2021).

#### Gene Ontology Term Functional Analysis

Because a high amount of the metaproteome (834 proteins) was common when different raw materials were used during the acetification, a GO Term analysis of exclusive proteins at each sampling time was performed to compare accurately the natural behavior of the microbiota. A total of 259 (FW_EL: 124; FW_UL: 135) and 200 (B_EL: 158; B_UL: 42) exclusive proteins were identified and detailed in Supplementary File 3. As previously mentioned, this analysis is mostly focused on the major amount of the metaproteome.

At the end of the loading phase for the fine wine profile (FW_EL), the metabolism of amino acids, mostly aminoacyl-tRNA ligases, cell division, and, briefly, stress-related response (metabolism of glutathione and chaperones) was highlighted between most abundant species. At the end of the exhausting phase (FW_UL), the formation of peptide release factors, ribosomal subunits, and stress-related response (chaperones, redox activity, and synthesis of lipopolysaccharides) were some of the most reported GO Terms. The end of the loading phase for the beer profile (B_EL) showed the metabolism of amino acids, energy metabolism pathways, and redox activity as main functions; exclusively identified in *K. europaeus*, outer membrane proteins as ABC transporters, and porins. At the end of the exhausting phase (B_UL), the predominant species were involved in ATP-binding, redox processes, and cellular homeostasis while the minor fractions were in stress-response (chaperones). *K. europaeus* (73.5%) not only shared the main GO terms with other related and minor species but was involved in other exclusive ones. A quantitative proteomic description of this species could provide an accurate approach to the microbiota function under the comparison of two acetification profiles.

### Comparative of Two Quantitative Proteomic Profiles in *Komagataeibacter europaeus*

**FIGURE 2:**
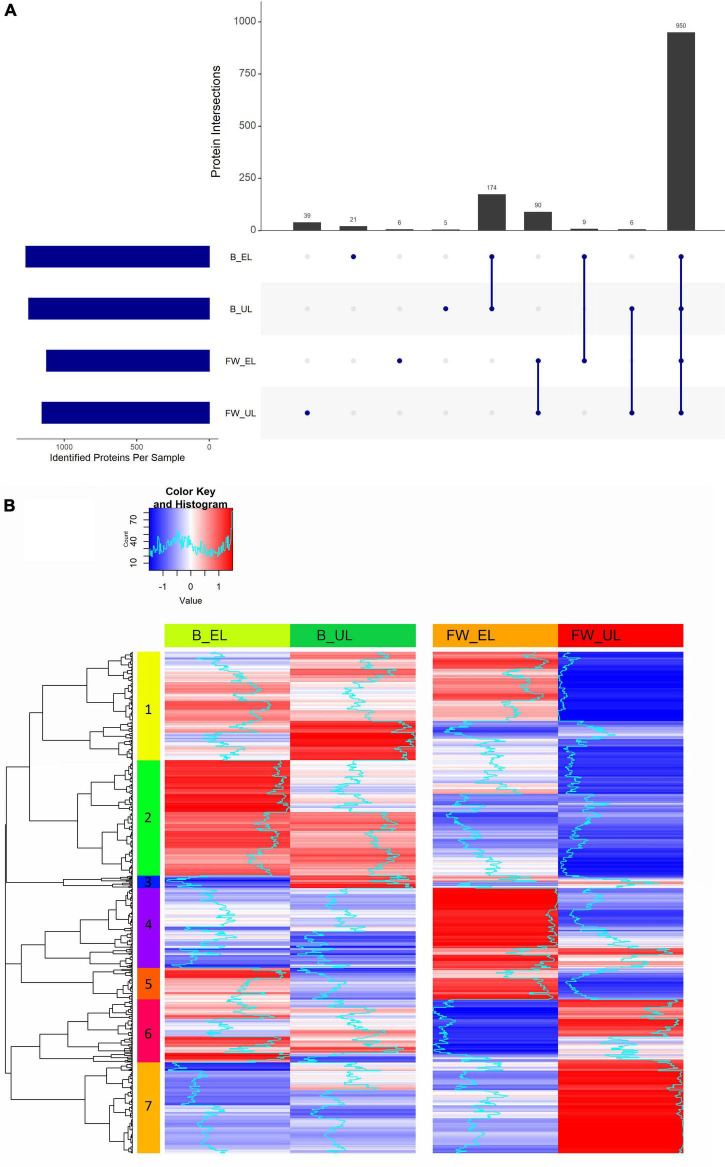
**(A)** Intersection diagram of the proteins of *K. europaeus* identified in at least 50% of total samples in at least one phase. The number of proteins from each intersection group is represented on the bars. **(B)** Heat map showing the analysis of hierarchical clustering of the proteins identified in at least 50% of total samples in each phase. The number on each color represents each of the seven built clusters. Fine wine (FW), beer (B), end of loading (EL), just before unloading (UL).

After subjecting all the samples to an LC/MS^2^ enrichment analysis, a total of 1,533 valid proteins were identified in *K. europaeus*. From them, 1,420 (B_EL: 1,264; B_UL: 1,245; FW_EL: 1,121; FW_UL: 1,152) were found in at least 50% of samples in one cycle time. The distribution of these proteins throughout the phases of two acetification profiles was summarized in an intersection plot shown in Figure 2A. Of the 1,420 proteins, 950 (66.9%) were common throughout both profiles, with 174 (12.3%) exclusive of the beer profile, and 90 (6.3%) of the fine wine profile. The amount of exclusive proteins at each sampling time was considerably minor: 39 out of 1,420 (2.7%) exclusive proteins at FW_UL were highlighted against 21 (1.5%) at B_EL, 6 (0.4%) at FW_EL, and 5 (0.4%) at B_UL. Then, 9 out of 1,420 (0.6%) proteins were found exclusively at the end of the loading phase (EL) and 6 (0.4%) before unloading (UL). The results evidenced that an important amount of the *K. europaeus* proteome was stable not affected by the change of phase or raw material.

#### Protein Clustering Analysis: Quantification Patterns and Interaction Networks

The proteome of *K. europaeus* was grouped according to the quantification pattern of each protein throughout each acetification profile. First, each protein quantification value in at least 50% of samples in all sampling times was normalized by z-score transformation and then clustered according to its pattern. Figure 2B shows a heatmap that summarized the hierarchical clustering carried out including a total of 832 proteins classified into seven clusters with different quantification patterns (more details can be found in Supplementary Table 2). Cluster 1 (*n* = 180) was characterized by a changing pattern throughout acetification of beer, but a marked decrease at the end of the exhausting phase in the fine wine vinegar (FW_UL). Cluster 2 (*n* = 191) was increased at the end of the loading phases, above all at B_EL, where quantification peaks were observed. Cluster 3 (*n* = 21), composed of a poor number of proteins, showed quantification peaks at B_UL. Clusters 4 (*n* = 132) and 5 (*n* = 53) were strongly upregulated at FW_EL, and also, Cluster 4 was decreased in the beer profile. Cluster 6 (*n* = 104) showed a changing pattern in the beer profile while in the fine wine profile, an increase just before unloading (FW_UL) was appreciated as in Cluster 7 (*n* = 151), where the quantification peaks were strongly observed (FW_UL).

**FIGURE 3:**
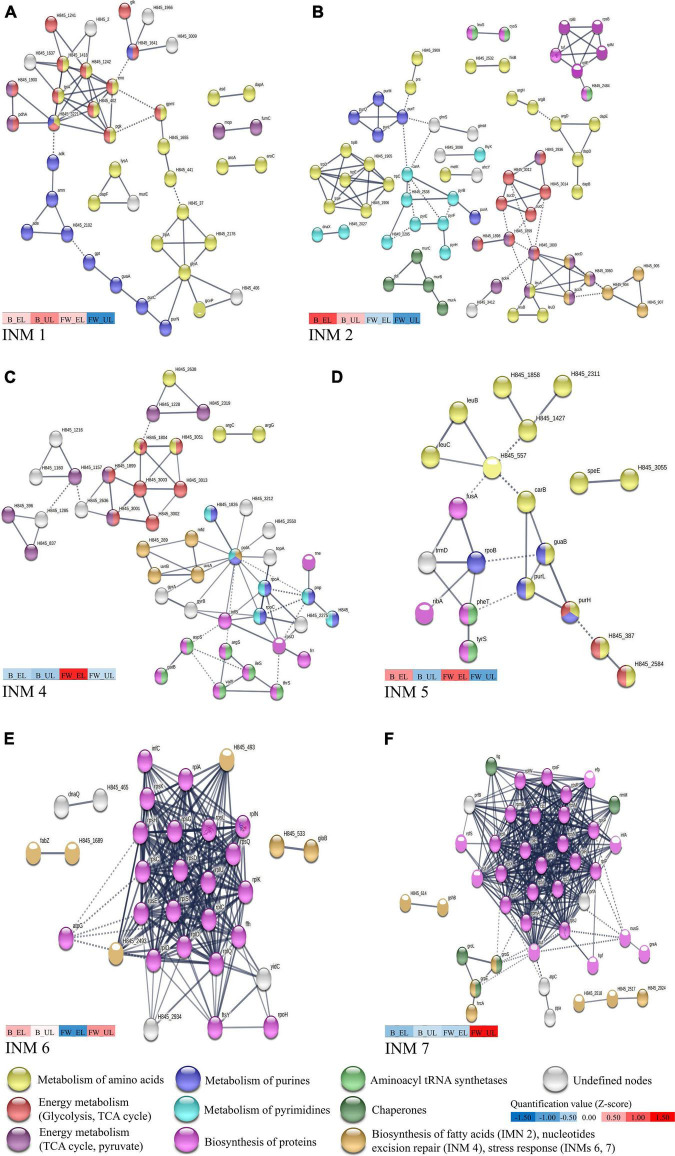
High confidence protein-protein interaction network maps (INM) performed in *K. europaeus* of proteins from each cluster, shown in Figure 2B, with a PPI enrichment *p*-value < 0.05 using STRING v11.0. **(A)** INM 1 (Cluster 1), **(B)** INM 2 (Cluster 2), **(C)** INM 4 (Cluster 4), **(D)** INM 5 (Cluster 5), **(E)** INM 6 (Cluster 6), and **(F)** INM 7 (Cluster 7). Proteins are shown as nodes and interactions between them are represented by edges whose thickness indicates the strength of each interaction. Nodes with the same color represent a specific function based on protein annotations according to the databases Uniprot and KEGG. A color scale showing the mean quantification values (z-score) of clustered proteins (see Figure 2B) that compose each INM at each sampling time is represented. Undefined nodes (gray color) belong to proteins with a low prevalent or unknown function. *K. xylinus* E25 was used as a model organism due to the high homology (MUM index of 0.21 with the genome of *K. europaeus*; Ryngajłło et al., 2018). The list of total proteins subjected to STRING analysis and their respective annotations can be found in Supplementary File 2.

Proteins from each cluster were then subjected to a protein-protein interaction analysis using the database STRING v11.0 to clarify the most relevant metabolic pathways related to each quantification pattern. Figure 3 shows INM built from each cluster (six out of seven are represented), and those showed more interactions than expected (PPI enrichment *p*-value < 0.05):

◼ INM 1 (82 edges; PPI enrichment *p*-value, 1.71 × 10^–13^) (Figure 3A) showed a high number of proteins related to the biosynthesis of amino acids (yellow nodes), mostly, L-glycine, L-serine, L-threonine, and L-lysine. A group of proteins at the top-left exhibited these proteins also involved in energy metabolism pathways [glycolysis (red nodes) and TCA cycle (purple nodes)]. Proteins related to the metabolism of purines (blue nodes) were found attached to it. Also, most of the alcohol dehydrogenase [ADH] subunits were classified in Cluster 1, even interaction groups that were not built (see Supplementary Table 2).
◼ INM 2 (123 edges; PPI enrichment *p*-value, 1.83 × 10^–07^) (Figure 3B) exhibited a high number of proteins for the biosynthesis of aromatic amino acids (yellow nodes) (L-phenylalanine, L-tryptophane, and L-tyrosine), see middle-left group. Proteins related to the TCA cycle (red nodes) and the pyruvate metabolism (purple nodes) were also shown and connected to fatty acid biosynthesis proteins (light brown nodes), see middle and bottom-right groups. The metabolism of purines (blue nodes) and pyrimidines (light blue nodes) were observed evidencing a similarity to INM 1, although some particular groups were appreciated as biosynthesis of peptidoglycans (dark green nodes) and proteins (pink nodes), see bottom-left and top-right groups.
◼ INM 3 was not built because did not reach a PPI enrichment *p*-value < 0.05.
◼ INM 4 (86 edges; PPI enrichment *p*-value, 1.77 × 10^–04^) (Figure 3C), similar to the previous INMs, showed groups relating processes like metabolism of amino acids (yellow nodes) to energy metabolism [TCA cycle (red nodes), pyruvate pathway (purple nodes)] and the purine (blue nodes) to pyrimidine metabolism (blue light nodes). Particularly, a group relating the biosynthesis of proteins (pink nodes) to aminoacyl-tRNA ligases (green nodes) was seen at the down-right.
◼ INM 5 (28 edges; PPI enrichment *p*-value, 2.95 × 10^–4^) (Figure 3D) following the trend of previous INMs, predominated biosynthesis of amino acids (yellow nodes) (L-alanine, L-aspartate, and L-glutamate), proteins (pink nodes), metabolism of purines (blue nodes), and aminoacyl-tRNA ligases (green nodes).
◼ INM 6 (227 edges; PPI enrichment *p*-value, 1.00 × 10^–16^) (Figure 3E) and INM 7 (353 edges; PPI enrichment *p*-value, 1.00 × 10^–16^) (Figure 3F) presented both a highlighted central group composed mainly of ribosomal subunits, initiation, and elongation factors (pink nodes); ribosomal silencing and maturation factors were also found in INM 7. Around and/or attached to the central protein group, chaperones (dark green nodes), stress-response proteins (oxidoreductases, metabolism of glutathione, aldehyde dehydrogenase [ALDH] subunits, and outer membrane efflux pumps) (light brown nodes) were represented.

#### Differential Expression Analysis by Pairs: ANOVA and HSD Tukey’s Test

**FIGURE 4:**
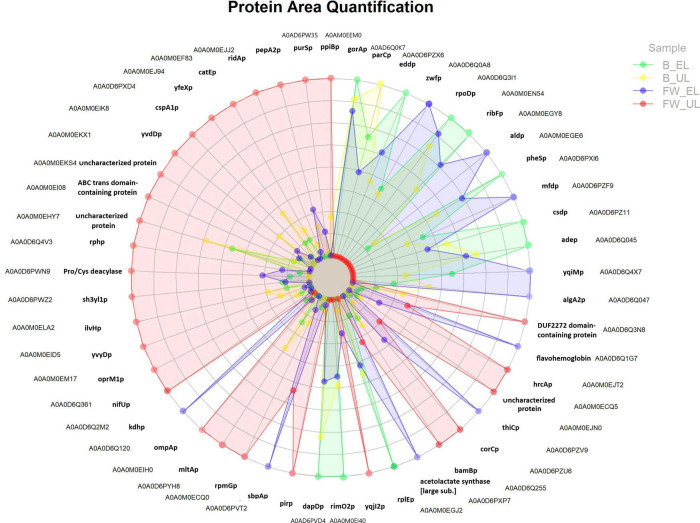
Radar chart representing proteins showing the strongest significative differences according to HSD Tukey’s test corrected by multiple testing (*q*-value < 0.01) and log_2_ fold change (in absolute value) > 2 (see Supplementary Table 3). The quantification area of each protein in each profile (FW, fine wine; B, beer) and sampling time (EL, end of loading; UL, just before unloading) is included.

A total of 141 proteins surpassed the statistical cut-off evidencing significant differences of quantification values in at least one pair comparison according to HSD Tukey’s test corrected by multiple testing (*q*-value < 0.05) and log_2_ fold change in absolute value (FC) > 1: one protein for the pair B_UL/B_EL, 23 proteins for the pair FW_EL/B_EL, 76 for the pair FW_UL/B_UL, and 108 for FW_UL/FW_EL (see Supplementary Table 3). From these proteins, those that showed a strong significance (Tukey corrected by *q*-value < 0.01 and FC > 2) were represented in a radar chart and will be described below (see Figure 4). This Figure is an easy way to visualize the relationship between proteins and their abundance in each sample so that it can be quickly seen that the protein profile depends on both the sampling time (EL, UL) and the raw material (FW, B). Then, a detailed description of these protein groups can be carried out.

First, proteins that exhibited quantification peaks at B_EL (green) were mostly shown at the top-right of the radar chart. As observed in the corresponding clusters and INMs (1 and 2; see Figures 2B, 3A,B) these proteins were involved in the metabolism of amino acids [glutathione reductase (gorAp), N-succinyl-transferase (dapDp)], of purines [adenine deaminase (adep)], aminoacyl-tRNA ligases [phenylalanine-tRNA ligase (pheSp)], and biosynthesis of proteins [riboflavin biosynthesis protein (ribFp), RNA polymerase sigma factor [RPOD] (rpoDp)]. Between them, rpoDp (cluster 1) was strongly down-regulated in the pairs FW_UL/B_UL and FW_UL/FW_EL (FC ≈ 3). One single protein presented the quantification peak at B_UL (yellow), DNA topoisomerase IV (parCp; cluster 1), essential in the segregation of chromosomes during DNA replication, especially down-regulated in the pair FW_UL/B_UL (FC ≈ 3).

Next, many proteins showed quantification peaks at FW_EL (blue), see clusters and INMs 4 and 5 (Figures 2B, 3C,D): acetolactate synthase [large subunit] [ALS], involved in the biosynthesis of branched-chain amino acids (BCAA); other proteins related to energy metabolism activity like the pentose phosphate pathway (PPP) [glucose-6-phosphate dehydrogenase [GPDH] (zwfp)] and the TCA cycle [α-ketoglutarate dehydrogenase [αKDH] (kdhp), 3-succinoyl-semialdehyde dehydrogenase [SSADH] (aldp)]; flavohemoglobin and flavin oxidoreductase [NADH] (yqiMp), known to be involved in the biosynthesis of flavoproteins that catalyzes oxidoreduction processes while sulfate-binding protein (sbpAp) and phospho-methyl-pyrimidine synthase [THIC] (thiCp) may provide FeS clusters for the electron transport chain. Most of these proteins were upregulated in the pair FW_EL/B_EL and downregulated in FW_UL/FW_EL; for the first one, thiCp was remarked (FC = 4.68). Although the aforementioned proteins maintained acceptable levels of expression in the phases described (B_EL, B_UL, and FW_EL), all of them were characterized by a significant decrease at FW_UL (red).

A total of 28 proteins, mostly distributed at the half left of the radar chart (see Figure 4), were characterized by significant quantification peaks at FW_UL (red) against a marked decrease in the rest of the phases, see clusters and INMs 6 and 7 (Figures 2B, 3E,F), and most of them were upregulated in FW_UL/B_UL and FW_UL/FW_EL pairs. Nitrogen-fixing thioredoxin (nifUp), iron-binding nuclear pirin (pirp), dehydrogenase PQQ (bamBp), and glyoxalase resistance protein (catEp) are related to oxidoreductase activity and maintain the redox balance. The outer membrane proteins ompAp and oprM1p, acting as porin of small solutes and efflux transport pump, respectively, and both playing a role in the outer membrane stability and resistance to environmental stress, were upregulated in the pair FW_UL/FW_EL (FC = 3.64 and 3.13 respectively). Other transmembrane proteins as ABC transporter (FC = 4.17) and murein hydrolase A [MLTA] (mltAp) (FC = 2.60) were then upregulated in the aforementioned pair while other proteins were related to the regulation of the translation: endoribonuclease [L-PSP] (ridAp), ribonuclease [RPH] (rphp), ribosome hibernation promoting factor [HPF] (yvyDp), and cold-shock protein [CSPA] (cspA1p). Acetolactate synthase [ILVH] (ilvHp) was shown, as occurred at FW_EL, while phospho-ribosylformylglycinamidine synthase [PURS] (purSp) catalyzed the first steps of the biosynthesis *de novo* of purines, and was one of the most upregulated proteins (FC = 5.58).

## Discussion

This study focused on the analysis of the acetification of two different substrates, aimed to delve into the behavior of the bacteria responsible for the process when the nutritional profile of the medium offers significant differences due to its composition, especially, in regard to the availability of carbon sources additional to ethanol, namely carbohydrates. Indeed, if the microbiota responsible is able to adapt to the conditions of the environment by modifying its metabolism and taking advantage of the resources available in each case, it would be a proof of its great versatility and therefore, survive in different and particularly, aggressive environments. In previous studies by the authors, qualitative and quantitative proteomic analysis of one synthetic alcoholic medium acetification process were carried out. Now, a new study is being conducted for much more complex media (fine wine and a craft beer) from which the differences in proteomic profiles are being disclosed. Then, a novelty from this study is that even when using different raw materials, the microbiota composition is similar, but its metabolism, at a proteome level, is different. Next, a detailed discussion about these issues will be carried out while the main differences between both profiles and approached proposals about the metabolic differences are made.

As it is known in the vinegar industry, the total strength of the medium (ethanol plus acetic acid concentration), which remains constant throughout the cycle, can affect to the cell activity and concentration (García-García et al., 2007; Baena-Ruano et al., 2010). In the present study, a mild environment offering no special stressing conditions has been used to study some basic aspects of the complex microbiota of the process. Here, both media showed an initial ethanol concentration of around 10% (v/v), and the acetic acid level could be disregarded (see section “Raw Material”). The initial total strength [% (w/v) of acetic acid plus % (v/v) of ethanol] is 10 total degrees. Then, in each cycle, 3.5/4 L of medium containing 9.2 [7.9 ± 0.2% (w/v) plus 1.3 ± 0.3% (v/v)] and 8.0 [6.8 ± 0.7% (w/v) plus 1.2 ± 0.1% (v/v)] total degrees for fine wine and beer profiles, respectively, are unloaded. The differences between the initial total strength and unloaded product appear due to the volatile losses (around 8 and 20% in each medium, respectively). The foaming generated in the beer medium would favor the volatile losses (20%) and no special care was taken to avoid these losses since it would not affect the aim of the work. Regardless, 92 and 80% of the disappeared ethanol was used for acetic acid formation and the rest was stripped by air or transformed by bacteria for other uses (Jiménez-Hornero et al., 2020).

Regarding the protein composition of the microbiota, no relevant differences were appreciated between the sampling times of each acetification profile. This composition showed a strong similarity to results obtained in our previous work that characterized an alcohol vinegar profile (Román-Camacho et al., 2021). These results may be explained by the operating mode followed in this work in which the same starter culture, consisting of a mixed broth coming from the aforementioned alcohol medium acetification, concretely, from the end of the ethanol exhausting phase, was used for both acetification profiles. Under these working conditions, in which the fine wine and then, beer acetification were consecutively performed, the raw material change might not modify excessively the starter microbial composition despite the additional nutritional richness that these natural substrates might provide. Subsequently, the main functions of the exclusive proteins in each phase were detailed to compare the microbiota activity in each acetification profile. The predominant species of the microbiota exhibited a natural behavior according to other authors that worked using submerged biotransformation (Fernández-Pérez et al., 2010; Qi et al., 2014; Trček et al., 2016) while the minor species showed a high-stress response, probably trying to coexist along with the better-adapted ones. Even if the protein amount that provides each species affects its role in the metaproteome, all of them might participate in the whole function of the microbial community (Peng et al., 2021). *K. europaeus*, supplying a mean frequency of 73.5%, far above the rest, not only shared the main GO Terms with other species but was involved in other exclusive ones. It is worth noting that this species was also the most representative in our previous studies (Román-Camacho et al., 2020, 2021). Therefore, a quantitative proteomic description of *K. europaeus*, comparing two acetification profiles, might provide a prediction of the microbiota role and characterize the natural raw materials used.

**FIGURE 5:**
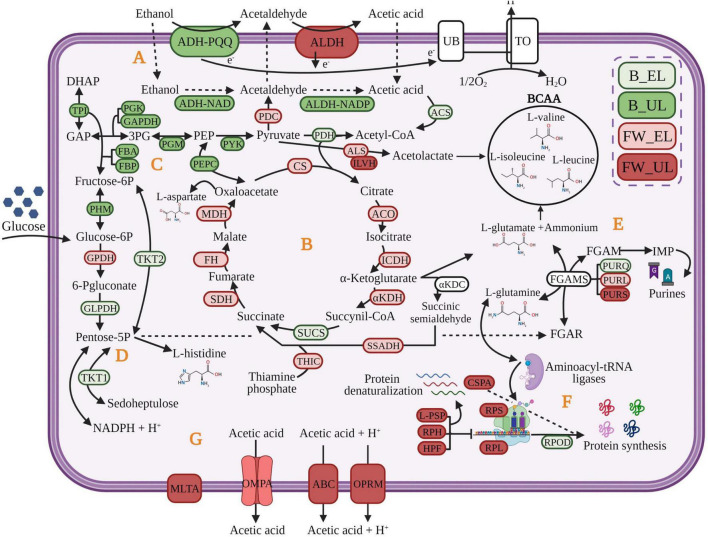
Molecular strategy of *K. europaeus* proposed to prevail throughout acetification process. The colors of the proteins represent the phase in which the protein had the highest quantification value. Fine wine (FW), beer (B), end of loading (EL), just before unloading (UL). **(A)** oxidation of ethanol into acetic acid; **(B)** TCA cycle; **(C)** glycolysis; **(D)** pentose phosphate pathway; **(E)**, amino acids and purines formation from biosynthetic precursors; **(F)** regulation of protein synthesis; **(G)** membrane mechanisms for acetic acid release. GAP, glyceraldehyde 3-phosphate; DHAP, dihydroxyacetone phosphate; 3PG, 3-phosphoglycerate; PEP, phosphoenolpyruvate; FGAR, formylglycinamide ribonucleotide; FGAM, formylglycinamidine ribonucleotide; IMP, inosine monophosphate. The definitions of the protein abbreviations and their respective highest quantification values are given (see Supplementary Table 4). Created with BioRender.com (with publication license).

*K. europaeus* is well-known as one of the main microorganisms responsible for industrial vinegar production. High ethanol-oxidizing ability, acetic acid requirement, and tolerance to high acidity levels [10–20% (w/v)] determine its suitability for this biotransformation (Trček et al., 2007; Yamada et al., 2012; Gullo et al., 2014). These capabilities allow it to perform an efficient incomplete oxidation reaction of the ethanol into acetic acid. This particular metabolic process consists of a two-step reaction (see Figure 5). First, alcohol dehydrogenase (ADH) binds to pyrroloquinoline quinone (PQQ) to oxidize the ethanol into acetaldehyde. Next, acetaldehyde is oxidized to acetic acid by aldehyde dehydrogenase (ALDH); both enzymes are located on the periplasmic side of the inner cell membrane (Adachi et al., 1980; Ameyama and Adachi, 1982). Further, NAD^+^ and NADP^+^ may be used as coenzymes by ADH-NAD and ALDH-NADP, located in the cytoplasm (Qin et al., 2021; Sriherfyna et al., 2021). The acetic acid produced at the periplasm is released into the medium increasing its external concentration which, in turn, triggers its diffusion and accumulation in the cytoplasm (Gullo et al., 2014; Qiu et al., 2021). The TCA cycle may assimilate the inner acetic acid through the input of acetyl-CoA providing biosynthetic precursors of amino acids and nucleic acids thus replenishing cell material throughout the loading phase and early stages of the ethanol depletion phase. The use of raw material with sugar content, as is the case of our craft beer, can lead to assimilating firstly, the available glucose and draining biosynthetic precursors directly from energy metabolic pathways as the PPP and the glycolysis. At the final moments of acetification, cells would trigger different membrane mechanisms dependent on proton motive force for the acetic acid release and detoxification. This molecular strategy, proposed in the present work, would allow *K. europaeus* to prevail over other species during the acetification process. These findings will be exhaustively detailed in the rest of the discussion based on hierarchical clustering, protein-protein interactions, and statistical analysis. Furthermore, it has been sectioned to facilitate the understanding of these microbial behavioral aspects at a quantitative level. In short, the discussion has been organized by analyzing the results obtained for the two raw materials as a whole according to the most relevant metabolic processes; and in this way, the differences existing in both acetification profiles can be better appreciated.

### The Essential Role of the Biosynthesis of Amino Acids and Nucleic Acids From Metabolic Precursors Replenishing Cellular Material Losses

The metabolism of amino acids seems to be one of the most representative metabolic pathways of *K. europaeus*, as can be appreciated in most protein clusters, above all, in those showing quantification peaks at the end of the loading phase (Clusters 1, 2, 4, and 5). The amino acids are synthesized from intermediaries of the TCA cycle, the glycolysis, and the PPP through L-glutamate and L-glutamine, both acting as nitrogen sources that are self-regulated according to the cell requirements (Yin et al., 2017; Sankuan et al., 2020). These results suggest that AAB might use their high nitrogen recovery capability to convert continuously nitrogen sources like proteins, nucleic acids derived from raw materials, and apoptotic cells into amino acids and ammonium thus replacing their consumption and cell material losses during the loading phase (Álvarez-Cáliz et al., 2012; Kuypers et al., 2018). In this sense, ALS is related to the synthesis of BCAA and L-valine, L-leucine, and L-isoleucine, from pyruvate, are strongly upregulated at FW_EL. It is also interesting to note that ILVH was highly upregulated at FW_UL. BCAA may provide NH_3_ and energy to neutralize the increase of acid final products during the exhausting phase and support intracellular pH balance through deamination, as proposed by other authors who reported different isoforms of acetolactate synthase in diverse acidophilic organisms (Santiago et al., 2012; Andrés-Barrao et al., 2016; Yin et al., 2017). Our findings supported the essential role of the metabolism of amino acids throughout acetification and especially, suggest the addition of BCAA to the fermentation culture as a possible system to protect the cellular integrity and increase productivity.

Conversely, the biosynthesis *de novo* of purines and pyrimidines requires the addition of amino acids to the pentose-5-phosphate, coming from the PPP, and metabolic energy (ATP) as can be observed in INMs belonging to clusters showing quantification peaks at EL (1, 2, 4, and 5). To our knowledge, this pathway has been barely researched in AAB, but here, phosphoribosyl-formyl-glycinamidine synthase complex (FGAMS) has been found (see Supplementary Table 2). FGAMS, composed of three subunits (PURQ, PURL, and PURS), carries out the ATP-dependent formation of formyl-glycinamidine ribonucleotide [FGAM] converting L-glutamine to L-glutamate (Tanwar et al., 2012). In previous works characterizing an alcohol vinegar microbiota (Román-Camacho et al., 2020, 2021), some GO Terms related to the synthesis of organic heterocyclic aromatic compounds, concretely, nucleic acids were identified. In the present work, the main subunits showed quantification peaks at different phases (PURQ, B_EL; PURL, FW_EL; PURS, FW_UL) although along with others, most of them were highlighted at the end of the loading phase. This fact could indicate that the synthesis of nucleic acids is integrated with other metabolic pathways, being part of a biological system that aims to replenish cellular material losses caused after unloading, improving adaptability, and ensuring the survival of the microbiota, above all, *K. europaeus*.

### The TCA Cycle as a Key Pathway in the Cytoplasmatic Acetic Acid Assimilation and Biosynthetic Precursors Source

The TCA cycle has been studied exhibiting an important function in the metabolism of AAB (Nakano and Fukaya, 2008; Kwong et al., 2017). The protein groups involved in this pathway were predominant in clusters whose quantification patterns were higher at EL phases (Clusters 1, 2, and 4). All the TCA cycle enzymes were found in the proteome of *K. europaeus* (see Supplementary Table 2) and all of them were downregulated, mainly in the fine wine profile (see Figure 5). In this work, αKDH, SSADH, and THIC were highlighted so they might play a critical role. Zhang and Bryant (2011) and Lei et al. (2018) investigated that αKDC and SSADH might form succinic acid via succinic semialdehyde by using cofactors of thiamine phosphate in *Synechococcus* sp. PCC7002. The TCA cycle can supply α-ketoglutarate to the synthesis of L-glutamate that provides amino groups in biosynthetic reactions, besides oxalacetate. For this purpose, the acetyl-CoA is provided to the TCA cycle by the conversion of pyruvate obtained in the glycolysis and of acetic acid derived from the ethanol oxidation (Mamlouk and Gullo, 2013; Qin et al., 2021). Because of the direct drain of intermediates from the TCA cycle to biomass, amino acids are partly derived from ethanol (Adler et al., 2014). In this sense, considering that AAB must cope with constant changes in ethanol, acetic acid, and cellular concentration because of the semi-continuous state of the cycles in our experiment, we suggest that the TCA cycle might be used for assimilating cytoplasmatic acetic acid, coming from ethanol, supplying energy, and biosynthetic precursors according to other authors (Ramírez-Bahena et al., 2013; Adler et al., 2014; Andrés-Barrao et al., 2016; Zheng et al., 2017).

### The Pentose Phosphate Pathway and Glycolysis Are Used to Assimilate the Available Glucose Obtaining Rapidly Biomass and Energy for the Synthesis of Precursors

The pentose phosphate pathway (PPP) is the main metabolic route of AAB to incompletely oxidize the glucose of the medium providing several precursor metabolites, mainly pentose-5-phosphate, necessary for the biosynthesis of amino acids (L-histidine) and nucleic acids (Adler et al., 2014; García-García et al., 2017). Several authors have related the enhance of PPP to the generation of NADPH + H^+^, also involved in biosynthetic processes even in reducing oxidative stress (Yin et al., 2017; Christodoulou et al., 2018; Sriherfyna et al., 2021). Although many of the species of *Acetobacter* and *Komagataeibacter* have demonstrated a higher preference for ethanol as a carbon source, in this analysis, most PPP enzymes were expressed when the ethanol concentration was higher (EL phases) (see Supplementary Table 2). Except for GPDH, which was strongly expressed in the fine wine profile (FW_EL), the rest of the PPP enzymes were higher quantified in the beer profile (B_EL) (see Figure 5). The remaining sugar content of the beer (7% before dilution) may provide glucose as a carbon source allowing AAB, mainly *K. europaeus*, to rapidly obtain biomass and energy for the synthesis of precursors (García-García et al., 2017; Qin et al., 2021). Indeed, the glycolysis enzymes were also higher expressed in the beer profile, in this case, and mostly upregulated (B_UL). Zheng et al. (2017) study showed that growing *A. pasteurianus* in a medium containing 1% initial acetic acid, PPP was decreased, and energy metabolism was enhanced by the production of pyruvate. Despite its ethanol preference, *K. europaeus* might assimilate, firstly, the glucose in the beer medium for rapid biosynthesis of precursors, which are not possible to obtain by other pathways, obtaining energy, and thus, prevail over other species that exhibit high glucose preference. This fact would explain the presence in our results of numerous protein groups involved in these pathways in clusters whose patterns showed quantification peaks during beer acetification (Clusters 1, 2, 4, and 5).

### The Biosynthesis of Ribosomes and Proteins Is Regulated With the Increase of Acetic Acid Concentration

The biosynthesis of proteins has been reported by different authors as one of the most highlighted metabolic pathways in AAB throughout the acetification process (Andrés-Barrao et al., 2012; Xia et al., 2016; Román-Camacho et al., 2021). Here, the most of functional groups involved in this process, mainly composed of ribosomal subunits, showed quantification peaks at FW_UL (Clusters 6 and 7). However, few of them were significantly upregulated since proteins that surpassed the statistical cut-off were related to the regulation of the translation. Among them, L-PSP inhibits the synthesis of proteins by the degradation of mRNAs, HPF dimerizes the bacterial functional ribosomes into inactive 100S ribosomes (Matzov et al., 2019), and RPH assists the maturation of tRNAs and the degradation of structured RNAs mainly in *E. coli* (Jain, 2012). Some authors have reported a decrease in the biosynthesis of proteins when the acidity levels increase in *A. pasteurianus* through functions as the recycling of ribosomes (Andrés-Barrao et al., 2012; Xia et al., 2016). Then, CSPA (see Figure 5), was strongly upregulated and its function has been discussed in *E. coli* as an RNA chaperone that prevents the protein refolding by ribonucleases (Rennella et al., 2017). When the acetic acid concentration is diluted during the loading phase the process of protein synthesis seems to occur efficiently through the presence of aminoacyl-tRNA ligases in Clusters 2, 4, and 5, binding tRNAs to specific amino acids and ensuring an accurate translation process (Román-Camacho et al., 2021). It is worth noting the drastic decrease of expression of initiation factors as RPOD with the increase of acetic acid level (FW_UL). In summary, these findings suggest that acetic acid accumulation generates a stress response thus regulating the formation of ribosomes and proteins.

### Membrane Mechanisms of Response to Acetic Acid Stress Derived From the Incomplete Oxidation of Ethanol

The incomplete oxidation of the ethanol of the medium is carried out by membrane-bound systems directly coupled to respiratory chains and allowing that oxidation reaction to take place in the periplasm without a requirement of transport across the membrane (Qin et al., 2021; Qiu et al., 2021). In this work, numerous subunits of PQQ-ADH and ALDH were identified and mainly expressed at UL phases (see Supplementary Table 2), but in general, these enzymes were stable indicating that the oxidation of ethanol could be constantly active throughout acetification. The acetic acid produced in the periplasm is released into the medium, thus increasing its external concentration. However, when it occurs, this compound can diffuse and accumulate into the cytoplasm along with that generated inside by the activity of ADH-NAD and ALDH-NADP (Adler et al., 2014; Gullo et al., 2014). In this sense, some systems acting to detoxify the cell might be participating through the upregulated proteins at FW_UL. First, those related to redox homeostasis maintenance, particularly, implicated the maturation of iron-sulfur (FeS) clusters acting as cofactors in the electron transfer (nifUp), cell apoptosis (pirp), and detoxification (catEp) (Benoit et al., 2018). To our knowledge, this set of proteins had never been reported in AAB. Secondly, outer membrane proteins, as permeable porins of small solutes (OMPA) and efflux pumps (OPRM, putative ABC-transporter) (see Figure 5), might control the cellular output of acetic acid, whose concentration increases during the fermentation phase (Nakano and Fukaya, 2008; Confer and Ayalew, 2013). MLTA, participating in the maintenance of the peptidoglycan layer under these conditions, show even more evidence of the importance of the cell surface as an efficient mechanism against the acetic acid stress used by the species of *Komagataeibacter*, as other authors have well-studied (Wang et al., 2015; Andrés-Barrao et al., 2016).

## Conclusion

A comparison of two acetification profiles using different raw materials was established to study the natural behavior of the involved microbiota through the metaproteome and, exhaustively, since it is the prevalent species, the quantitative proteomic profile of *K. europaeus*. Although the use of different raw materials seems not to affect the microbial composition, the microbiota behaved differently by significant changes in the expression of the proteome. In this work, it has been suggested that the inner acetic acid coming from the oxidation of ethanol might be assimilated in the TCA cycle providing biosynthetic precursors along with other metabolic pathways (PPP and glycolysis) if glucose is available, as is the case of one of the fermentation media studied (craft beer). These processes replenish the cell material losses (amino acids and nucleic acids) after unloading, throughout the loading phase. The excess of acetic acid in the cytoplasm would also be released to the medium by some cell membrane mechanisms proton motive-force dependent at the final stages of the acetification. This complete strategy has been reported in Figure 5, highlighting the phase at which each protein had a higher quantification value. The differences in the metabolic behavior throughout each acetification profile were more accentuated in the fine wine vinegar than in the craft beer vinegar. In this profile, FW_UL was a period significantly differentially based on statistical analysis. Metabolomic assays that would allow clarifying the differences between the associated metabolites to these raw materials, in more detail, are underway. These findings may lay the groundwork of a vinegar microbiota profile, at a protein level, under smooth operating conditions. Future studies might be undertaken to evaluate the effect on the microbiota of media with higher levels of ethanol and acetic acid and even comparatives studies to achieve a multi-omics integrative profile. This work might increase the knowledge of the use of diverse raw materials and optimize the operating conditions.

## Data Availability Statement

The datasets presented in this study can be found in online repositories. The names of the repository/repositories and accession number(s) can be found below: NCBI—PXD031147.

## Author Contributions

JR-C: methodology, validation, formal analysis, data curation, writing—original draft preparation, and visualization. JM and IG-G: conceptualization, investigation, resources, writing—review and editing, supervision, project administration, and funding acquisition. IS-D: methodology, validation, formal analysis, conceptualization, and visualization. TG-M: conceptualization, data curation, validation, and supervision. All authors contributed to the article and approved the submitted version.

## Conflict of Interest

The authors declare that the research was conducted in the absence of any commercial or financial relationships that could be construed as a potential conflict of interest.

## Publisher’s Note

All claims expressed in this article are solely those of the authors and do not necessarily represent those of their affiliated organizations, or those of the publisher, the editors and the reviewers. Any product that may be evaluated in this article, or claim that may be made by its manufacturer, is not guaranteed or endorsed by the publisher.

## Footnotes

1
http://www.uniprot.org

2
http://geneontology.org/

3
https://string-db.org/

4
https://www.genome.jp/kegg/

## References

[B1] Adachi O. Tayama K. Shinagawa E. Matsushita K. Ameyama M. (1980). Purification and characterization of membrane-bound aldehyde dehydrogenase from Gluconobacter suboxydans. *Agric. Biol. Chem.* 44 503–515. 10.1271/bbb1961.44.503

[B2] Adler P. Frey L. J. Berger A. Bolten C. J. Hansen C. E. Wittmann C. (2014). The key to acetate: metabolic fluxes of *acetic acid bacteria* under cocoa pulp fermentation-simulating conditions. *Appl. Environ. Microbiol.* 80 4702–4716. 10.1128/AEM.01048-14 24837393 PMC4148806

[B3] Álvarez-Cáliz C. Santos-Dueñas I. M. Cañete-Rodríguez A. M. García-Martínez T. Mauricio J. C. García-García I. (2012). Free amino acids, urea and ammonium ion contents for submerged wine vinegar production: influence of loading rate and air-flow rate. *Acetic Acid Bact.* 1:e1. 10.4081/aab.2012.e1

[B4] Ameyama M. Adachi O. (1982). “Alcohol dehydrogenase from *acetic acid bacteria*, membrane bound,” in *Methods in Enzymology*, ed. Wood W. A. (New York: Academic Press), 450–457. 10.1016/S0076-6879(82)89078-2

[B5] Andrés-Barrao C. Saad M. M. Cabello-Ferrete E. Bravo D. Chappuis M. L. Ortega-Pérez R. (2016). Metaproteomics and ultrastructure characterization of Komagataeibacter spp. involved in high-acid spirit vinegar production. *Food Microbiol.* 55 112–122. 10.1016/j.fm.2015.10.012 26742622

[B6] Andrés-Barrao C. Saad M. M. Chappuis M. L. Boffa M. Perret X. Ortega-Pérez R. (2012). Proteome analysis of Acetobacter pasteurianus during acetic acid fermentation. *J. Proteome* 75 1701–1717. 10.1016/j.jprot.2011.11.027 22155126

[B7] Baena-Ruano S. Jiménez-Ot C. Santos-Dueñas I. M. Cantero-Moreno D. Barja F. García-García I. (2006). Rapid method for total, viable and non-viable *acetic acid bacteria* determination during acetification process. *Process Biochem.* 41 1160–1164. 10.1016/j.procbio.2005.12.016

[B8] Baena-Ruano S. Jiménez-Ot C. Santos-Dueñas I. M. Jiménez-Hornero J. E. Bonilla-Venceslada J. L. Álvarez-Cáliz C. (2010). Influence of the final ethanol concentration on the acetification and production rate in the wine vinegar process. *J. Chem. Technol. Biotechnol.* 85 908–912. 10.1002/jctb.2368

[B9] Benoit S. L. Holland A. A. Johnson M. K. Maier R. J. (2018). Iron–sulfur protein maturation in *Helicobacter* pylori: identifying a Nfu-type cluster carrier protein and its iron–sulfur protein targets. *Mol. Microbiol.* 108 379–396. 10.1111/mmi.13942 29498770 PMC5943153

[B10] Bradford M. M. (1976). A rapid and sensitive method for the quantitation of microgram quantities of protein utilizing the principle of protein dye binding. *Anal. Biochem.* 72 248–254. 10.1016/0003-2697(76)90527-3 942051

[B11] Christodoulou D. Link H. Fuhrer T. Kochanowski K. Gerosa L. Sauer U. (2018). Reserve flux capacity in the pentose phosphate pathway enables *Escherichia coli*’s rapid response to oxidative stress. *Cell Syst.* 6 569–578. 10.1016/j.cels.2018.04.009 29753645

[B12] Confer A. W. Ayalew S. (2013). The OmpA family of proteins: roles in bacterial pathogenesis and immunity. *Vet. Microbiol.* 163 207–222. 10.1016/j.vetmic.2012.08.019 22986056

[B13] Fernández-Pérez R. Torres C. Sanz S. Ruiz-Larrea F. (2010). Strain typing of *acetic acid bacteria* responsible for vinegar production by the submerged elaboration method. *Food Microbiol.* 27 973–978. 10.1016/j.fm.2010.05.020 20832673

[B14] García-García I. Cañete-Rodríguez A. M. Santos-Dueñas I. M. Jiménez-Hornero J. E. Ehrenreich A. Liebl W. (2017). Biotechnologically relevant features of gluconic acid production by *acetic acid bacteria*. *Acetic Acid Bact.* 6:6458. 10.4081/aab.2017.6458

[B15] García-García I. Cantero-Moreno D. Jiménez-Ot C. Baena-Ruano S. Jiménez-Hornero J. Santos-Dueñas I. (2007). Estimating the mean acetification rate via on-line monitored changes in ethanol during a semi-continuous vinegar production cycle. *J. Food Eng.* 80 460–464. 10.1016/j.jfoodeng.2006.05.028

[B16] García-García I. Jiménez-Hornero J. E. Santos-Dueñas I. M. González-Granados Z. Cañete-Rodríguez A. M. (2019). “Modeling and optimization of acetic acid fermentation,” in *Advances in vinegar production*, ed. Bekatorou A. (Madrid, Spain: Taylor & Francis Group: CRC Press), 10.1201/9781351208475

[B17] Gullo M. Verzelloni E. Canonico M. (2014). Aerobic submerged fermentation by *acetic acid bacteria* for vinegar production: process and biotechnological aspects. *Process Biochem.* 49 1571–1579. 10.1016/j.procbio.2014.07.003

[B18] Hidalgo C. Torija M. J. Mas A. Mateo E. (2013). Effect of inoculation on strawberry fermentation and acetification processes using native strains of yeast and *acetic acid bacteria*. *Food Microbiol.* 34 88–94. 10.1016/j.fm.2012.11.019 23498182

[B19] Jain C. (2012). Novel role for RNase PH in the degradation of structured RNA. *J. Bacteriol.* 194 3883–3890. 10.1128/JB.06554-11 22609921 PMC3416528

[B20] Jiang Y. Lv X. Zhang C. Zheng Y. Zheng B. Duan X. (2019). Microbial dynamics and flavor formation during the traditional brewing of Monascus vinegar. *Food Res. Int.* 125:108531. 10.1016/j.foodres.2019.108531 31554138

[B21] Jiménez-Hornero J. E. Santos-Dueñas I. M. García-García I. (2020). Modelling acetification with Artificial Neural Networks and comparison with alternative procedures. *Processes* 8:749. 10.3390/pr8070749

[B22] Kandylis P. Bekatorou A. Dimitrellou D. Plioni I. Giannopoulou K. (2021). Health Promoting Properties of Cereal Vinegars. *Foods* 10:344. 10.3390/foods10020344 33562762 PMC7914830

[B23] Kuypers M. Marchant H. Kartal B. (2018). The microbial nitrogen-cycling network. *Nat. Rev. Microbiol.* 16 263–276. 10.1038/nrmicro.2018.9 29398704

[B24] Kwong W. Zheng H. Moran N. (2017). Convergent evolution of a modified, acetate-driven TCA cycle in bacteria. *Nat. Microbiol.* 2:17067. 10.1038/nmicrobiol.2017.67 28452983 PMC5482284

[B25] Lei G. Wang X. Lai C. Li Z. M. Zhang W. Xie C. (2018). Expression and biochemical characterization of α-ketoglutarate decarboxylase from cyanobacterium Synechococcus sp. PCC7002. *Int. J. Biol. Macromol.* 114 188–193. 10.1016/j.ijbiomac.2018.03.112 29574001

[B26] Li S. Li P. Feng F. Luo L. X. (2015). Microbial diversity and their roles in the vinegar fermentation process. *Appl. Microbiol. Biotechnol.* 99 4997–5024. 10.1007/s00253-015-6659-1 25971198

[B27] Llaguno C. (1991). “Definición y tipos de vinagre,” in *El vinagre de vino*, eds Llaguno C. Polo M. C. (Madrid, Spain: Consejo Superior de Investigaciones Científicas (CSIC)), 133–145.

[B28] Mamlouk D. Gullo M. (2013). *Acetic acid bacteria*: physiology and carbon sources oxidation. *Indian J. Microbiol.* 53 377–384. 10.1007/s12088-013-0414-z 24426139 PMC3779290

[B29] Mas A. Torija M. J. García-Parrilla M. C. Troncoso A. M. (2014). *Acetic acid bacteria* and the production and quality of wine vinegar. *Sci. World J.* 2014:394671. 10.1155/2014/394671 24574887 PMC3918346

[B30] Matzov D. Bashan A. Yap M. N. F. Yonath A. (2019). Stress response as implemented by hibernating ribosomes: a structural overview. *FEBS J.* 286 3558–3565. 10.1111/febs.14968 31230411 PMC6746590

[B31] Nakano S. Fukaya M. (2008). Analysis of proteins responsive to acetic acid in Acetobacter: molecular mechanisms conferring acetic acid resistance in *acetic acid bacteria*. *Int. J. Food Microbiol.* 125 54–59. 10.1016/j.ijfoodmicro.2007.05.015 17920150

[B32] Peng M. Y. Zhang X. J. Huang T. Zhong X. Z. Chai L. J. Lu Z. M. (2021). Komagataeibacter europaeus improves community stability and function in solid-state cereal vinegar fermentation ecosystem: Non-abundant species plays an important role. *Food Res. Int.* 150:110815. 10.1016/j.foodres.2021.110815 34863491

[B33] Qi Z. Yang H. Xia X. Quan W. Wang W. Yu X. (2014). Achieving high strength vinegar fermentation via regulating cellular growth status and aeration strategy. *Process Biochem.* 49 1063–1070. 10.1016/j.procbio.2014.03.018

[B34] Qin Z. Yu S. Chen J. Zhou J. (2021). Dehydrogenases of *acetic acid bacteria*. *Biotechnol. Adv.* 54:107863. 10.1016/j.biotechadv.2021.107863 34793881

[B35] Qiu X. Zhang Y. Hong H. (2021). Classification of *acetic acid bacteria* and their acid resistant mechanism. *AMB Expr.* 11:29. 10.1186/s13568-021-01189-6 33595734 PMC7889782

[B36] Ramírez-Bahena M. H. Tejedor C. Martín I. Velázquez E. Peix A. (2013). Endobacter medicaginis gen. nov., sp. nov., isolated from alfalfa nodules in an acidic soil. *Int. J. Syst. Evol. Microbiol.* 63 1760–1765. 10.1099/ijs.0.041368-0 23002052

[B37] Rennella E. Sára T. Juen M. Wunderlich C. Imbert L. Solyom Z. (2017). RNA binding and chaperone activity of the E. coli cold-shock protein CspA. *Nucleic Acids Res.* 45 4255–4268. 10.1093/nar/gkx044 28126922 PMC5397153

[B38] Román-Camacho J. J. Mauricio J. C. Santos-Dueñas I. M. García-Martínez T. García-García I. (2021). Functional metaproteomic analysis of alcohol vinegar microbiota during an acetification process: a quantitative proteomic approach. *Food Microbiol.* 98:103799. 10.1016/j.fm.2021.103799 33875225

[B39] Román-Camacho J. J. Santos-Dueñas I. M. García-García I. Moreno-García J. García-Martínez T. Mauricio J. C. (2020). Metaproteomics of microbiota involved in submerged culture production of alcohol wine vinegar: a first approach. *Int. J. Food Microbiol.* 333:108797. 10.1016/j.ijfoodmicro.2020.108797 32738750

[B40] Ryngajłło M. Kubiak K. Jędrzejczak-Krzepkowska M. Jacek P. Bielecki S. (2018). Comparative genomics of the Komagataeibacter strains–Efficient bionanocellulose producers. *Microbiologyopen* 8:e00731. 10.1002/mbo3.731 30365246 PMC6528568

[B41] Sankuan X. Cuimei Z. Bingqian F. Yu Z. Menglei X. Linna T. (2020). Metabolic network of ammonium in cereal vinegar solid-state fermentation and its response to acid stress. *Food Microbiol.* 95:103684. 10.1016/j.fm.2020.103684 33397616

[B42] Santiago B. MacGilvray M. Faustoferri R. C. Quivey R. G. Jr. (2012). The branched-chain amino acid aminotransferase encoded by ilvE is involved in acid tolerance in Streptococcus mutans. *J. Bacteriol.* 194 2010–2019. 10.1128/JB.06737-11 22328677 PMC3318461

[B43] Sriherfyna F. H. Matsutani M. Hirano K. Koike H. Kataoka N. Yamashita T. (2021). The auxiliary NADH dehydrogenase plays a crucial role in redox homeostasis of nicotinamide cofactors in the absence of the periplasmic oxidation system in Gluconobacter oxydans NBRC3293. *Appl. Environ. Microbiol.* 87 e02155–20. 10.1128/AEM.02155-20 33127815 PMC7783338

[B44] Tanwar A. S. Morar M. Panjikar S. Anand R. (2012). Formylglycinamide ribonucleotide amidotransferase from *Salmonella* typhimurium: role of ATP complexation and the glutaminase domain in catalytic coupling. *Acta Crystallogr. D* 68 627–636. 10.1107/S0907444912006543 22683785

[B45] Trček J. Jernejc K. Matsushita K. (2007). The highly tolerant acetic acid bacterium Gluconacetobacter europaeus adapts to the presence of acetic acid by changes in lipid composition, morphological properties and PQQ-dependent ADH expression. *Extremophiles* 11 627–635. 10.1007/s00792-007-0077-y 17487444

[B46] Trček J. Mahnič A. Rupnik M. (2016). Diversity of the microbiota involved in wine and organic apple cider submerged vinegar production as revealed by DHPLC analysis and next-generation sequencing. *Int. J. Food Microbiol.* 223 57–62. 10.1016/j.ijfoodmicro.2016.02.007 26897250

[B47] Verce M. De Vuyst L. Weckx S. (2019). Shotgun Metagenomics of a Water Kefir Fermentation Ecosystem Reveals a Novel Oenococcus Species. *Front. Microbiol.* 10:479. 10.3389/fmicb.2019.00479 30918501 PMC6424877

[B48] Wang B. Shao Y. Chen F. (2015). Overview on mechanisms of acetic acid resistance in *acetic acid bacteria*. *World J. Microbiol. Biotechnol.* 31 255–263. 10.1007/s11274-015-1799-0 25575804

[B49] Xia K. Zang N. Zhang J. Zhang H. Li Y. Liu Y. (2016). New insights into the mechanisms of acetic acid resistance in Acetobacter pasteurianus using iTRAQ-dependent quantitative proteomic analysis. *Int. J. Food Microbiol.* 238 241–251. 10.1016/j.ijfoodmicro.2016.09.016 27681379

[B50] Yamada Y. Yukphan P. Lan-Vu H. T. Muramatsu Y. Ochaikul D. Tanasupawat S. (2012). Description of Komagataeibacter gen. nov., with proposals of new combinations (*Acetobacteraceae*). *J. Gen. Appl. Microbiol.* 58 397–404. 10.2323/jgam.58.397 23149685

[B51] Yin H. Zhang R. Xia M. Bai X. Mou J. Zheng Y. (2017). Effect of aspartic acid and glutamate on metabolism and acid stress resistance of Acetobacter pasteurianus. *Microb. Cell Factories* 16:109. 10.1186/s12934-017-0717-6 28619110 PMC5472864

[B52] Zhang S. Bryant D. A. (2011). The Tricarboxylic Acid Cycle in Cyanobacteria. *Science* 334 1551–1553. 10.1126/science.1210858 22174252

[B53] Zhang X. Wang P. Dandan X. Wang W. Zhao Y. (2019). Aroma patterns of Beijing rice vinegar and their potential biomarker for traditional Chinese cereal vinegars. *Food Res. Int.* 119 398–410. 10.1016/j.foodres.2019.02.008 30884670

[B54] Zheng Y. Zhang R. Yin H. Bai X. Chang Y. Xia M. (2017). Acetobacter pasteurianus metabolic change induced by initial acetic acid to adapt to acetic acid fermentation conditions. *Appl. Microbiol. Biotechnol.* 101 7007–7016. 10.1007/s00253-017-8453-8 28770302

[B55] Zhu Y. Zhang F. Zhang C. Yang L. Fan G. Xu Y. (2018). Dynamic microbial succession of Shanxi aged vinegar and its correlation with flavor metabolites during different stages of acetic acid fermentation. *Sci. Rep.* 8:8612. 10.1038/s41598-018-26787-6 29872163 PMC5988729

