# Supplementary material for: Unraveling the Role of Acetic Acid Bacteria Comparing Two Acetification Profiles From Natural Raw Materials: A Quantitative Approach in Komagataeibacter europaeus
Source: Front Microbiol. 2022 Apr 29;13:840119. doi: 10.3389/fmicb.2022.840119 (PMC9100681; doi:10.3389/fmicb.2022.840119)
Supplement: Supplementary file 1 [file Presentation_1.pdf]

Supplementary Material

1 Supplementary Data

File S1. List of raw data containing the total proteins identified in the LC/MS-MS analysis for the vinegar samples of fine wine and beer in at least 50% out of the total in at least one sampling time, as well as those of the starter inoculum used. Several parameters of each protein are included as accession number (Uniprot), quantification value of each sample, organism, score, emPAI, protein name, and gene name, among others.

File S2. List of total proteins that compose each of the INM (Interaction network map), those with a PPI enrichment *p*-value < 0.05. The node code, the identifier, and annotated functions of network proteins based on the databases Uniprot (http://www.uniprot.org) and KEGG (https://www.genome.jp/kegg/) are included.

File S3. List of exclusive proteins identified in the LC/MS-MS analysis in fine wine and beer vinegar profiles. The exclusive proteins were obtained by the difference between those in at least 50% out of total samples in at least one sampling time and those in each sampling time (EL, end of loading; UL, just before unloading). Several parameters of each protein are included as accession number (Uniprot), quantification value of each sample, organism, score, emPAI, protein name, and gene name, among others.

2 Supplementary Figures and Tables

2.1 Supplementary Tables

Table S1. Microbial composition of the metaproteome identified by LC-MS/MS analysis. The frequency (%) is represented as the number of proteins provided by each species in each profile (FW, fine wine; B, beer) and sampling time (EL, end of loading; UL, just before unloading) out of the total proteins. The cumulative frequency of the species and the mean frequencies are included.

Table S2. List of proteins of *K. europaeus* identified in at least 50% out of total samples in each profile (FW, fine wine; B, beer) and sampling time (EL, end of loading; UL, just before unloading). The accession number (Uniprot), protein name, description, cluster to which each protein belongs, and z-score quantification values are included.

Table S3. List of total proteins that surpassed the statistical cut-off in at least one out of four pairs comparison (B_UL/B_EL, FW_EL/B_EL, FW_UL/B_UL, FW_UL/FW_EL) according to HSD

Tukey´s test corrected by multiple testing (*q*-value < 0.05) and $\log_2$ fold change in absolute value > 1. * = medium significance, ** = high significance.

Table S4. List of abbreviations whose proteins participate in the molecular strategy of *K. europaeus* proposed in Fig. 5. The protein name, accession number (Uniprot), the highest quantification value, and its corresponding phase are given.
