# Supplementary material for: Unraveling the Role of Acetic Acid Bacteria Comparing Two Acetification Profiles From Natural Raw Materials: A Quantitative Approach in Komagataeibacter europaeus
Source: Front Microbiol. 2022 Apr 29;13:840119. doi: 10.3389/fmicb.2022.840119 (PMC9100681; doi:10.3389/fmicb.2022.840119)
Supplement: Supplementary file 2 [file Table_1.docx]

| Table S1. Microbial composition of the metaproteome identified by LC-MS/MS analysis. The frequency (%) is represented as the number of proteins provided by each species in each profile (FW, fine wine; B, beer) and sampling time (EL, end of loading; UL, just before unloading) out of the total proteins. The cumulative frequency of the species and the mean frequencies are included. | | | | | | | | | | | | | | | |
| --- | --- | --- | --- | --- | --- | --- | --- | --- | --- | --- | --- | --- | --- | --- | --- |
|  | Number of proteins | |  |  | Frequency out of total proteins (%) | | | | Mean freq. (%) | Cumulative frequency (%) | | |  | Mean cumulative freq. (%) |  |
| Species | FW_EL | FW_UL | B_EL | B_UL | FW_EL | FW_UL | B_EL | B_UL |  | FW_EL | FW_UL | B_EL | B_UL |  |  |
| *Komagataeibacter europaeus* | 709 | 709 | 865 | 806 | 75.9 | 75.0 | 70.6 | 72.6 | 73.5 | 75.9 | 75.0 | 70.6 | 72.6 | 73.5 |  |
| *Komagataeibacter xylinus* | 33 | 27 | 54 | 47 | 3.5 | 2.9 | 4.4 | 4.2 | 3.8 | 79.4 | 77.9 | 75.0 | 76.8 | 77.3 |  |
| *Komagataeibacter intermedius* | 22 | 20 | 25 | 21 | 2.4 | 2.1 | 2.0 | 1.9 | 2.1 | 81.8 | 80.0 | 77.0 | 78.7 | 79.4 |  |
| *Komagataeibacter rhaeticus* | 19 | 20 | 26 | 22 | 2.0 | 2.1 | 2.1 | 2.0 | 2.1 | 83.8 | 82.1 | 79.1 | 80.7 | 81.4 |  |
| *Komagataeibacter diospyri* | 16 | 14 | 22 | 18 | 1.7 | 1.5 | 1.8 | 1.6 | 1.7 | 85.5 | 83.6 | 80.9 | 82.3 | 83.1 |  |
| *Komagataeibacter swingsii* | 12 | 15 | 21 | 18 | 1.3 | 1.6 | 1.7 | 1.6 | 1.6 | 86.8 | 85.2 | 82.6 | 84.0 | 84.7 |  |
| *Komagataeibacter medellinensis* | 11 | 8 | 10 | 7 | 1.2 | 0.8 | 0.8 | 0.6 | 0.9 | 88.0 | 86.0 | 83.4 | 84.6 | 85.5 |  |
| *Komagataeibacter nataicola* | 4 | 7 | 13 | 10 | 0.4 | 0.7 | 1.1 | 0.9 | 0.8 | 88.4 | 86.8 | 84.5 | 85.5 | 86.3 |  |
| *Komagataeibacter oboediens* | 7 | 7 | 12 | 7 | 0.7 | 0.7 | 1.0 | 0.6 | 0.8 | 89.2 | 87.5 | 85.5 | 86.1 | 87.1 |  |
| *Acetobacter sp.* | 3 | 8 | 9 | 10 | 0.3 | 0.8 | 0.7 | 0.9 | 0.7 | 89.5 | 88.4 | 86.2 | 87.0 | 87.8 |  |
| *Komagataeibacter sp.* | 5 | 6 | 10 | 9 | 0.5 | 0.6 | 0.8 | 0.8 | 0.7 | 90.0 | 89.0 | 87.0 | 87.8 | 88.5 |  |
| *Gluconacetobacter sp.* | 8 | 5 | 7 | 9 | 0.9 | 0.5 | 0.6 | 0.8 | 0.7 | 90.9 | 89.5 | 87.6 | 88.6 | 89.2 |  |
| *Komagataeibacter sucrofermentans* | 5 | 3 | 11 | 10 | 0.5 | 0.3 | 0.9 | 0.9 | 0.7 | 91.4 | 89.8 | 88.5 | 89.5 | 89.8 |  |
| *Acetobacteraceae (unclassified)* | 2 | 4 | 7 | 4 | 0.2 | 0.4 | 0.6 | 0.4 | 0.4 | 91.6 | 90.3 | 89.1 | 89.9 | 90.2 |  |
| *Acidomonas methanolica* | 4 | 4 | 4 | 4 | 0.4 | 0.4 | 0.3 | 0.4 | 0.4 | 92.1 | 90.7 | 89.4 | 90.3 | 90.6 |  |
| *Acetobacter malorum* | 3 | 4 | 5 | 4 | 0.3 | 0.4 | 0.4 | 0.4 | 0.4 | 92.4 | 91.1 | 89.8 | 90.6 | 91.0 |  |
| *Acidisphaera rubrifaciens* | 3 | 3 | 5 | 4 | 0.3 | 0.3 | 0.4 | 0.4 | 0.4 | 92.7 | 91.4 | 90.2 | 91.0 | 91.3 |  |
| *Commensalibacter sp.* | 3 | 2 | 6 | 4 | 0.3 | 0.2 | 0.5 | 0.4 | 0.3 | 93.0 | 91.6 | 90.7 | 91.4 | 91.7 |  |
| *Gluconacetobacter diazotrophicus* | 3 | 2 | 6 | 4 | 0.3 | 0.2 | 0.5 | 0.4 | 0.3 | 93.4 | 91.9 | 91.2 | 91.7 | 92.0 |  |
| *Komagataeibacter saccharivorans* | 2 | 3 | 5 | 4 | 0.2 | 0.3 | 0.4 | 0.4 | 0.3 | 93.6 | 92.2 | 91.6 | 92.1 | 92.4 |  |
| *Acetobacter pasteurianus* | 2 | 5 | 3 | 3 | 0.2 | 0.5 | 0.2 | 0.3 | 0.3 | 93.8 | 92.7 | 91.8 | 92.3 | 92.7 |  |
| *Acidocella sp.* | 2 | 4 | 4 | 2 | 0.2 | 0.4 | 0.3 | 0.2 | 0.3 | 94.0 | 93.1 | 92.2 | 92.5 | 93.0 |  |
| *Gluconobacter thailandicus* | 5 | 4 | 1 | 1 | 0.5 | 0.4 | 0.1 | 0.1 | 0.3 | 94.5 | 93.5 | 92.3 | 92.6 | 93.2 |  |
| *Acetobacter pomorum* | 2 | 6 | 2 | 1 | 0.2 | 0.6 | 0.2 | 0.1 | 0.3 | 94.8 | 94.2 | 92.4 | 92.7 | 93.5 |  |
| *Komagataeibacter cocois* | 2 | 2 | 4 | 3 | 0.2 | 0.2 | 0.3 | 0.3 | 0.3 | 95.0 | 94.4 | 92.7 | 93.0 | 93.8 |  |
| *Rhodopila globiformis* | 2 | 2 | 4 | 3 | 0.2 | 0.2 | 0.3 | 0.3 | 0.3 | 95.2 | 94.6 | 93.1 | 93.2 | 94.0 |  |
| *Gluconacetobacter liquefaciens* | 1 | 1 | 4 | 5 | 0.1 | 0.1 | 0.3 | 0.5 | 0.2 | 95.3 | 94.7 | 93.4 | 93.7 | 94.3 |  |
| *Komagataeibacter pomaceti* | 3 | 2 | 3 | 2 | 0.3 | 0.2 | 0.2 | 0.2 | 0.2 | 95.6 | 94.9 | 93.6 | 93.9 | 94.5 |  |
| *Acetobacter tropicalis* | 2 | 2 | 3 | 3 | 0.2 | 0.2 | 0.2 | 0.3 | 0.2 | 95.8 | 95.1 | 93.9 | 94.1 | 94.7 |  |
| *Gluconacetobacter entanii* | 2 | 2 | 3 | 3 | 0.2 | 0.2 | 0.2 | 0.3 | 0.2 | 96.0 | 95.3 | 94.1 | 94.4 | 95.0 |  |
| *Gluconobacter oxydans* | 1 | 3 | 3 | 3 | 0.1 | 0.3 | 0.2 | 0.3 | 0.2 | 96.1 | 95.7 | 94.4 | 94.7 | 95.2 |  |
| *Neokomagataea tanensis* | 1 | 1 | 4 | 4 | 0.1 | 0.1 | 0.3 | 0.4 | 0.2 | 96.3 | 95.8 | 94.7 | 95.0 | 95.4 |  |
| *Acidocella aminolytica* | 2 | 2 | 3 | 2 | 0.2 | 0.2 | 0.2 | 0.2 | 0.2 | 96.5 | 96.0 | 94.9 | 95.2 | 95.7 |  |
| *Acetobacter ascendens* | 2 | 5 | 1 | 0 | 0.2 | 0.5 | 0.1 | 0.0 | 0.2 | 96.7 | 96.5 | 95.0 | 95.2 | 95.9 |  |
| *Acidiphilum sp.* | 1 | 1 | 4 | 3 | 0.1 | 0.1 | 0.3 | 0.3 | 0.2 | 96.8 | 96.6 | 95.4 | 95.5 | 96.1 |  |
| *Neoasaia chiangmaiensis* | 1 | 1 | 4 | 3 | 0.1 | 0.1 | 0.3 | 0.3 | 0.2 | 96.9 | 96.7 | 95.7 | 95.8 | 96.3 |  |
| *Komagataeibacter maltaceti* | 2 | 1 | 3 | 2 | 0.2 | 0.1 | 0.2 | 0.2 | 0.2 | 97.1 | 96.8 | 95.9 | 95.9 | 96.5 |  |
| *Acetobacter oeni* | 2 | 0 | 3 | 3 | 0.2 | 0.0 | 0.2 | 0.3 | 0.2 | 97.3 | 96.8 | 96.2 | 96.2 | 96.6 |  |
| *Acetobacter orientalis* | 1 | 2 | 2 | 2 | 0.1 | 0.2 | 0.2 | 0.2 | 0.2 | 97.4 | 97.0 | 96.3 | 96.4 | 96.8 |  |
| *Acetobacter cerevisiae* | 2 | 2 | 1 | 1 | 0.2 | 0.2 | 0.1 | 0.1 | 0.1 | 97.6 | 97.2 | 96.4 | 96.5 | 96.9 |  |
| *Dankookia rubra* | 1 | 1 | 2 | 2 | 0.1 | 0.1 | 0.2 | 0.2 | 0.1 | 97.8 | 97.4 | 96.6 | 96.7 | 97.1 |  |
| *Gluconobacter albidus* | 1 | 1 | 2 | 2 | 0.1 | 0.1 | 0.2 | 0.2 | 0.1 | 97.9 | 97.5 | 96.7 | 96.8 | 97.2 |  |
| *Komagataeibacter hansenii* | 1 | 1 | 2 | 2 | 0.1 | 0.1 | 0.2 | 0.2 | 0.1 | 98.0 | 97.6 | 96.9 | 97.0 | 97.4 |  |
| *Acetobacter cibinongensis* | 2 | 1 | 1 | 1 | 0.2 | 0.1 | 0.1 | 0.1 | 0.1 | 98.2 | 97.7 | 97.0 | 97.1 | 97.5 |  |
| *Oleomonas sp.* | 1 | 2 | 1 | 1 | 0.1 | 0.2 | 0.1 | 0.1 | 0.1 | 98.3 | 97.9 | 97.1 | 97.2 | 97.6 |  |
| *Gluconobacter cerinus* | 1 | 1 | 2 | 1 | 0.1 | 0.1 | 0.2 | 0.1 | 0.1 | 98.4 | 98.0 | 97.2 | 97.3 | 97.7 |  |
| *Commensalibacter intestini* | 1 | 0 | 2 | 2 | 0.1 | 0.0 | 0.2 | 0.2 | 0.1 | 98.5 | 98.0 | 97.4 | 97.5 | 97.8 |  |
| *Acetobacter senegalensis* | 0 | 0 | 3 | 2 | 0.0 | 0.0 | 0.2 | 0.2 | 0.1 | 98.5 | 98.0 | 97.6 | 97.7 | 97.9 |  |
| *Granulibacter bethesdensis* | 0 | 0 | 3 | 2 | 0.0 | 0.0 | 0.2 | 0.2 | 0.1 | 98.5 | 98.0 | 97.9 | 97.8 | 98.1 |  |
| *Neokomagataea sp.* | 0 | 0 | 3 | 2 | 0.0 | 0.0 | 0.2 | 0.2 | 0.1 | 98.5 | 98.0 | 98.1 | 98.0 | 98.2 |  |
| *Acetobacter aceti* | 1 | 2 | 1 | 0 | 0.1 | 0.2 | 0.1 | 0.0 | 0.1 | 98.6 | 98.2 | 98.2 | 98.0 | 98.3 |  |
| *Gluconobacter wancherniae* | 1 | 1 | 1 | 1 | 0.1 | 0.1 | 0.1 | 0.1 | 0.1 | 98.7 | 98.3 | 98.3 | 98.1 | 98.4 |  |
| *Parasaccharibacter apium* | 1 | 1 | 1 | 1 | 0.1 | 0.1 | 0.1 | 0.1 | 0.1 | 98.8 | 98.4 | 98.4 | 98.2 | 98.5 |  |
| *Roseococcus sp.* | 1 | 1 | 1 | 1 | 0.1 | 0.1 | 0.1 | 0.1 | 0.1 | 98.9 | 98.5 | 98.5 | 98.3 | 98.5 |  |
| *Acetobacter orleanensis* | 1 | 1 | 2 | 0 | 0.1 | 0.1 | 0.2 | 0.0 | 0.1 | 99.0 | 98.6 | 98.6 | 98.3 | 98.6 |  |
| *Asaia platycodi* | 1 | 0 | 1 | 2 | 0.1 | 0.0 | 0.1 | 0.2 | 0.1 | 99.1 | 98.6 | 98.7 | 98.5 | 98.7 |  |
| *Swingsia samuiensis* | 0 | 1 | 2 | 1 | 0.0 | 0.1 | 0.2 | 0.1 | 0.1 | 99.1 | 98.7 | 98.9 | 98.6 | 98.8 |  |
| *Gluconobacter sphaericus* | 1 | 2 | 0 | 0 | 0.1 | 0.2 | 0.0 | 0.0 | 0.1 | 99.3 | 98.9 | 98.9 | 98.6 | 98.9 |  |
| *Roseomonas sp.* | 1 | 2 | 0 | 0 | 0.1 | 0.2 | 0.0 | 0.0 | 0.1 | 99.4 | 99.2 | 98.9 | 98.6 | 99.0 |  |
| *Acetobacter okinawensis* | 0 | 1 | 0 | 2 | 0.0 | 0.1 | 0.0 | 0.2 | 0.1 | 99.4 | 99.3 | 98.9 | 98.7 | 99.1 |  |
| *Saccharibacter sp.* | 0 | 1 | 1 | 1 | 0.0 | 0.1 | 0.1 | 0.1 | 0.1 | 99.4 | 99.4 | 98.9 | 98.8 | 99.1 |  |
| *Acetobacter oryzoeni* | 1 | 1 | 0 | 0 | 0.1 | 0.1 | 0.0 | 0.0 | 0.1 | 99.5 | 99.5 | 98.9 | 98.8 | 99.2 |  |
| *Gluconobacter frateurii* | 1 | 1 | 0 | 0 | 0.1 | 0.1 | 0.0 | 0.0 | 0.1 | 99.6 | 99.6 | 98.9 | 98.8 | 99.2 |  |
| *Acetobacter persici* | 0 | 2 | 0 | 0 | 0.0 | 0.2 | 0.0 | 0.0 | 0.1 | 99.6 | 99.8 | 98.9 | 98.8 | 99.3 |  |
| *Roseomonas cervicalis* | 1 | 0 | 0 | 1 | 0.1 | 0.0 | 0.0 | 0.1 | 0.0 | 99.7 | 99.8 | 98.9 | 98.9 | 99.3 |  |
| *Acetobacter estunensis* | 0 | 1 | 0 | 1 | 0.0 | 0.1 | 0.0 | 0.1 | 0.0 | 99.7 | 99.9 | 98.9 | 99.0 | 99.4 |  |
| *Roseomonas rosea* | 1 | 0 | 1 | 0 | 0.1 | 0.0 | 0.1 | 0.0 | 0.0 | 99.8 | 99.9 | 99.0 | 99.0 | 99.4 |  |
| *Zavarzinia sp.* | 0 | 1 | 1 | 0 | 0.0 | 0.1 | 0.1 | 0.0 | 0.0 | 99.8 | 100 | 99.1 | 99.0 | 99.5 |  |
| *Acetobacter peroxydans* | 0 | 0 | 1 | 1 | 0.0 | 0.0 | 0.1 | 0.1 | 0.0 | 99.8 | 100 | 99.2 | 99.1 | 99.5 |  |
| *Kozakia baliensis* | 0 | 0 | 1 | 1 | 0.0 | 0.0 | 0.1 | 0.1 | 0.0 | 99.8 | 100 | 99.3 | 99.2 | 99.6 |  |
| *Rhodovastum atsumiense* | 0 | 0 | 1 | 1 | 0.0 | 0.0 | 0.1 | 0.1 | 0.0 | 99.8 | 100 | 99.3 | 99.3 | 99.6 |  |
| *Roseomonas gilardii* | 0 | 0 | 1 | 1 | 0.0 | 0.0 | 0.1 | 0.1 | 0.0 | 99.8 | 100 | 99.4 | 99.4 | 99.6 |  |
| *Stella humosa* | 0 | 0 | 1 | 1 | 0.0 | 0.0 | 0.1 | 0.1 | 0.0 | 99.8 | 100 | 99.5 | 99.5 | 99.7 |  |
| *Swaminathania salitolerans* | 0 | 0 | 1 | 1 | 0.0 | 0.0 | 0.1 | 0.1 | 0.0 | 99.8 | 100 | 99.6 | 99.5 | 99.7 |  |
| *Swingsia sp.* | 0 | 0 | 1 | 1 | 0.0 | 0.0 | 0.1 | 0.1 | 0.0 | 99.8 | 100 | 99.7 | 99.6 | 99.8 |  |
| *Acetobacter indonesiensis* | 0 | 0 | 2 | 0 | 0.0 | 0.0 | 0.2 | 0.0 | 0.0 | 99.8 | 100 | 99.8 | 99.6 | 99.8 |  |
| *Gluconobacer morbifer* | 1 | 0 | 0 | 0 | 0.1 | 0.0 | 0.0 | 0.0 | 0.0 | 99.9 | 100 | 99.8 | 99.6 | 99.8 |  |
| *Roseomonas deserti* | 1 | 0 | 0 | 0 | 0.1 | 0.0 | 0.0 | 0.0 | 0.0 | 100 | 100 | 99.8 | 99.6 | 99.9 |  |
| *Asaia bogorensis* | 0 | 0 | 0 | 1 | 0.0 | 0.0 | 0.0 | 0.1 | 0.0 | 100 | 100 | 99.8 | 99.7 | 99.9 |  |
| *Paracraurococcus sp.* | 0 | 0 | 0 | 1 | 0.0 | 0.0 | 0.0 | 0.1 | 0.0 | 100 | 100 | 99.8 | 99.8 | 99.9 |  |
| *Roseomonas oryzae* | 0 | 0 | 0 | 1 | 0.0 | 0.0 | 0.0 | 0.1 | 0.0 | 100 | 100 | 99.8 | 99.9 | 99.9 |  |
| *Zavarzinia compransoris* | 0 | 0 | 0 | 1 | 0.0 | 0.0 | 0.0 | 0.1 | 0.0 | 100 | 100 | 99.8 | 100 | 100 |  |
| *Gluconobacter kanchanaburiensis* | 0 | 0 | 1 | 0 | 0.0 | 0.0 | 0.1 | 0.0 | 0.0 | 100 | 100 | 99.9 | 100 | 100 |  |
| *Tanticharoenia sakaeratensis* | 0 | 0 | 1 | 0 | 0.0 | 0.0 | 0.1 | 0.0 | 0.0 | 100 | 100 | 100 | 100 | 100 |  |
| Total | 934 | 945 | 1226 | 1110 | 100 | 100 | 100 | 100 | 100 | 100 | 100 | 100 | 100 | 100 |  |
