# Supplementary material for: Unraveling the Role of Acetic Acid Bacteria Comparing Two Acetification Profiles From Natural Raw Materials: A Quantitative Approach in Komagataeibacter europaeus
Source: Front Microbiol. 2022 Apr 29;13:840119. doi: 10.3389/fmicb.2022.840119 (PMC9100681; doi:10.3389/fmicb.2022.840119)
Supplement: Supplementary file 4 [file Table_3.DOCX]

| Table S3. List of total proteins that surpassed the statistical cut-off in at least one out of four pairs comparison (B_UL/B_EL, FW_EL/B_EL, FW_UL/B_UL, FW_UL/FW_EL) according to HSD Tukey´s test corrected by multiple testing (*q*-value < 0.05) and log_2_ fold change in absolute value > 1. * = medium significance, ** = high significance. | | | | | | | | | | |
| --- | --- | --- | --- | --- | --- | --- | --- | --- | --- | --- |
|  |  |  | |  |  |  |  |  |  |  |
|  |  | | HDS Tukey´s test (corrected by *q*-value) | | | | Log _2_ fold change | | | |
| Accession number | Description | | B_UL/B_EL | FW_EL/B_EL | FW_UL/B_UL | FW_UL/FW_EL | B_UL/B_EL | FW_EL/B_EL | FW_UL/B_UL | FW_UL/FW_EL |
| A0A0D6PVA2 | Bifunctional uridylyl-transferase | | 1.000 | 0.123 | 1.000 | 0.013* |  |  |  | -1.41 |
| A0A0D6PVA6 | Threonine synthase | | 1.000 | 0.013* | 0.386 | 0.029* |  | 3.30 |  | -1.28 |
| A0A0D6PVD4 | N-succinyltransferase | | 1.000 | 0.166 | 0.035* | 0.190 |  |  | -2.59 |  |
| A0A0D6PVE5 | 3-hydroxyacyl dehydratase FabZ | | 1.000 | 0.463 | 0.129 | 0.011* |  |  |  | 1.65 |
| A0A0D6PVF0 | 1-deoxy-D-xylulose 5-P reductoisomerase | | 0.591 | 0.049* | 0.427 | 1.000 |  | -1.70 |  |  |
| A0A0D6PVT2 | Iron-binding nuclear pirin [pir] | | 1.000 | 1.000 | 0.004** | 0.002** |  |  | 3.33 | 3.85 |
| A0A0D6PW29 | ABC transporter HlyD/EmrA/FusE | | 0.539 | 0.022* | 0.396 | 0.025* |  | -2.48 |  | 2.22 |
| A0A0D6PW35 | Phospho-ribosylormyl-glycinamidine synthase [PurS] | | 1.000 | 1.000 | 0.015* | 0.009** |  |  | 5.58 | 2.81 |
| A0A0D6PWD4 | Metal dependent hydrolase | | 1.000 | 0.007** | 0.278 | 0.000** |  |  |  | -1.83 |
| A0A0D6PWG7 | M20_dimer domain-containing protein | | 1.000 | 0.357 | 0.028* | 0.256 |  |  | 3.67 |  |
| A0A0D6PWK8 | 50S ribosomal protein L27 | | 1.000 | 1.000 | 0.031* | 0.101 |  |  | 2.27 |  |
| A0A0D6PWM4 | Glutamate--tRNA ligase | | 1.000 | 0.325 | 0.004** | 0.035* |  |  | -1.23 |  |
| A0A0D6PWN9 | Cys-tRNA (Pro)/Cys-tRNA (Cys) deacylase | | 1.000 | 1.000 | 0.004** | 0.009** |  |  | 2.36 | 1.30 |
| A0A0D6PWZ2 | Ysc84 domain-containing protein | | 1.000 | 1.000 | 0.001** | 0.001** |  |  | 2.90 | 2.01 |
| A0A0D6PX21 | 10 kDa chaperonin | | 1.000 | 1.000 | 0.031* | 0.028* |  |  | 3.55 | 2.73 |
| A0A0D6PX25 | Acetyl-coenzyme A carboxylase sub. alpha | | 0.659 | 0.040* | 0.468 | 1.000 |  | -2.17 |  |  |
| A0A0D6PXD4 | Iron-dependent peroxidase | | 1.000 | 1.000 | 0.024* | 0.003** |  |  | 1.03 | 2.04 |
| A0A0D6PXD8 | Oligopeptidase B | | 1.000 | 0.725 | 0.305 | 0.016* |  |  |  | -1.60 |
| A0A0D6PXI6 | Phenylalanine--tRNA ligase alpha sub. | | 0.223 | 0.244 | 0.013* | 0.003** |  |  | -1.55 | -1.81 |
| A0A0D6PXV1 | 30S ribosomal protein S6 | | 1.000 | 1.000 | 0.013* | 0.014* |  |  | 1.84 | 1.34 |
| A0A0D6PXY2 | Oxidoreductase SDR | | 1.000 | 0.725 | 0.034* | 0.027* |  |  | -1.18 | -1.16 |
| A0A0D6PY79 | Threonine--tRNA ligase | | 1.000 | 0.040* | 0.395 | 0.002** |  |  |  | -1.22 |
| A0A0D6PYE3 | Nitrite/sulfite reductase | | 1.000 | 1.000 | 0.113 | 0.022* |  |  |  | -1.30 |
| A0A0D6PYG3 | Gamma-glutamyl phosphate reductase | | 1.000 | 0.458 | 0.065 | 0.005** |  |  |  | -1.44 |
| A0A0D6PYG8 | Endoribonuclease L-PSP | | 1.000 | 0.569 | 0.029* | 0.144 |  |  | 4.81 |  |
| A0A0D6PYH8 | 50S ribosomal protein L33 | | 1.000 | 1.000 | 0.035* | 0.012* |  |  | 2.18 | 2.80 |
| A0A0D6PYZ5 | Bifunctional protein GlmU | | 1.000 | 1.000 | 0.052 | 0.034* |  |  |  | -1.48 |
| A0A0D6PZB3 | P-glycerate mutase | | 0.596 | 1.000 | 0.021* | 0.401 |  |  | -1.75 |  |
| A0A0D6PZF5 | Cobaltochelatase cobS sub. | | 1.000 | 1.000 | 0.031* | 0.004** |  |  | -1.44 | -1.84 |
| A0A0D6PZF9 | Transcription-repair-coupling factor | | 1.000 | 0.108 | 0.345 | 0.005** |  |  |  | -2.39 |
| A0A0D6PZH2 | Amido-phosphoribosyl-transferase (ATase) | | 0.349 | 0.762 | 0.081 | 0.008** |  |  |  | -1.52 |
| A0A0D6PZU6 | Dehydrogenase PQQ | | 1.000 | 1.000 | 0.013* | 0.003** |  |  | 1.42 | 2.19 |
| A0A0D6PZV9 | Hemolysin/magnesium/cobalt transporter CorC/HlyC | | 1.000 | 0.584 | 0.021* | 0.056 |  |  | 3.05 |  |
| A0A0D6PZX6 | DNA topoisomerase 4 sub. A | | 0.591 | 0.630 | 0.003** | 0.029* |  |  | -3.07 | -2.21 |
| A0A0D6PZZ8 | 4-aminobutyrate aminotransferase | | 1.000 | 0.022* | 0.405 | 0.072 |  | 3.79 |  |  |
| A0A0D6Q014 | P-ribosyl-glycinamide formyl-transferase | | 1.000 | 1.000 | 0.129 | 0.027* |  |  |  | -1.36 |
| A0A0D6Q020 | Methionine--tRNA ligase | | 1.000 | 1.000 | 0.038* | 0.033* |  |  | -1.44 | -1.40 |
| A0A0D6Q045 | Adenine deaminase | | 1.000 | 0.411 | 0.035* | 0.036* |  |  | -1.75 | -1.66 |
| A0A0D6Q047 | Mannose-1-phosphate guanylyltransferase | | 1.000 | 0.023* | 0.133 | 0.003** |  | 1.25 |  | -2.01 |
| A0A0D6Q0A4 | UvrABC system protein A | | 1.000 | 0.155 | 0.260 | 0.014* |  |  |  | -1.93 |
| A0A0D6Q0I1 | Chaperone protein HtpG | | 1.000 | 0.032* | 1.000 | 0.013* |  | 1.83 |  | -1.87 |
| A0A0D6Q0K7 | Glutathione reductase | | 1.000 | 1.000 | 0.031* | 0.028* |  |  | -2.92 | -2.82 |
| A0A0D6Q0Y6 | Thioredoxin | | 1.000 | 1.000 | 0.042* | 0.062 |  |  | 1.76 |  |
| A0A0D6Q120 | Outer membrane protein OmpA [like-domain] | | 1.000 | 1.000 | 0.001** | 0.000** |  |  | 3.14 | 3.13 |
| A0A0D6Q1C7 | Alpha-acetolactate decarboxylase | | 1.000 | 0.029* | 0.809 | 0.029* |  | 4.64 |  | -2.11 |
| A0A0D6Q1G7 | Flavohemoglobin | | 1.000 | 0.001** | 0.04* | 0.002** |  | 2.80 | 2.11 | -1.34 |
| A0A0D6Q1K2 | Transcription termination/antitermination protein | | 1.000 | 1.000 | 0.003** | 0.002** |  |  | 1.39 | 1.37 |
| A0A0D6Q1M9 | ATP synthase gamma chain | | 1.000 | 0.040* | 1.000 | 0.101 |  | -1.70 |  |  |
| A0A0D6Q1N2 | Adenosyl-homocysteinase | | 1.000 | 0.262 | 0.027* | 0.482 |  |  | -1.59 |  |
| A0A0D6Q1V1 | Amidohydrolase 3 | | 1.000 | 0.058 | 0.970 | 0.024* |  |  |  | -2.74 |
| A0A0D6Q255 | Acetolactate synthase large sub. | | 1.000 | 0.009** | 0.313 | 0.008** |  | 2.52 |  | -1.60 |
| A0A0D6Q2C2 | Aminopeptidase | | 0.455 | 1.000 | 0.01* | 0.161 |  |  | -1.60 |  |
| A0A0D6Q2I3 | 5-aminolevulinate synthase | | 1.000 | 1.000 | 0.078 | 0.013* |  |  |  | -1.96 |
| A0A0D6Q2M2 | Alpha-ketoglutarate dehydrogenase | | 1.000 | 0.022* | 1.000 | 0.007** |  | 1.93 |  | -2.25 |
| A0A0D6Q2M9 | Flavin-dependent thymidylate synthase | | 0.223 | 0.880 | 0.04* | 0.003** |  |  | -1.23 | -1.73 |
| A0A0D6Q2N4 | Pyruvate kinase | | 0.197 | 1.000 | 0.015* | 1.000 |  |  | -1.20 |  |
| A0A0D6Q306 | Histidinol dehydrogenase | | 1.000 | 0.040* | 0.277 | 0.305 |  | 1.78 |  |  |
| A0A0D6Q361 | Nitrogen-fixing thioredoxin [NifU] | | 1.000 | 1.000 | 0.003** | 0.002** |  |  | 3.69 | 3.72 |
| A0A0D6Q387 | DNA helicase | | 1.000 | 0.068 | 0.031* | 0.004** |  |  |  | -1.15 |
| A0A0D6Q395 | Polyribonucleotide nucleotidyltransferase | | 1.000 | 0.058 | 0.970 | 0.005** |  |  |  | -1.33 |
| A0A0D6Q3C9 | Urocanate hydratase | | 1.000 | 0.040* | 0.723 | 0.118 |  | 2.60 |  |  |
| A0A0D6Q3I1 | Glucose-6-phosphate 1-dehydrogenase | | 1.000 | 0.081 | 0.164 | 0.004** |  |  |  | -2.88 |
| A0A0D6Q3K3 | Isocitrate dehydrogenase [NADP] | | 1.000 | 1.000 | 0.056 | 0.019* |  |  |  | -1.70 |
| A0A0D6Q3N8 | DUF2272 domain-containing protein | | 1.000 | 1.000 | 0.051 | 0.011* |  |  |  | 3.22 |
| A0A0D6Q4V3 | Ribonuclease PH | | 1.000 | 1.000 | 0.01* | 0.011* |  |  | 2.22 | 1.58 |
| A0A0D6Q4X7 | Flavin oxidoreductase [NADH] | | 0.776 | 0.088 | 0.260 | 0.002** |  |  |  | -2.62 |
| A0A0M0ECK7 | Virginiamycin B lyase | | 1.000 | 0.081 | 0.997 | 0.003** |  |  |  | -1.02 |
| A0A0M0ECQ0 | Sulfate-binding protein | | 1.000 | 0.003* | 0.022* | 0.017* |  | 2.58 | 2.29 |  |
| A0A0M0ECQ5 | Uncharacterized protein | | 1.000 | 1.000 | 0.009** | 0.003** |  |  | 2.11 | 2.58 |
| A0A0M0ECS4 | Chromosome-partitioning ATPase Soj | | 1.000 | 0.040* | 1.000 | 0.062 |  | -1.32 |  |  |
| A0A0M0ECT6 | Uncharacterized protein | | 1.000 | 0.015* | 0.027* | 0.481 |  | 5.20 | 4.20 |  |
| A0A0M0EDB3 | Dihydroxy-acid dehydratase | | 1.000 | 0.123 | 1.000 | 0.022* |  |  |  | -1.56 |
| A0A0M0EEG8 | Inosine-5'-monophosphate dehydrogenase | | 0.565 | 0.210 | 0.330 | 0.002** |  |  |  | -1.69 |
| A0A0M0EEL6 | Glyceraldehyde-3-P dehydrogenase | | 0.565 | 0.269 | 0.015* | 1.000 |  |  | -1.88 |  |
| A0A0M0EEM0 | Peptidyl-prolyl cis-trans isomerase | | 1.000 | 1.000 | 0.015* | 0.003** |  |  | 1.41 | 2.32 |
| A0A0M0EEN4 | Phosphoglycerate kinase | | 0.565 | 0.960 | 0.013* | 0.430 |  |  | -2.34 |  |
| A0A0M0EF24 | Triosephosphate isomerase | | 1.000 | 1.000 | 0.024* | 0.101 |  |  | -1.82 |  |
| A0A0M0EF83 | Endoribonuclease L-PSP | | 1.000 | 1.000 | 0.003** | 0.002** |  |  | 3.26 | 4.01 |
| A0A0M0EFE7 | Ribose-phosphate pyrophosphokinase | | 0.006** | 0.010* | 0.048* | 0.005** | -1.05 |  |  | -1.23 |
| A0A0M0EFG0 | Protein RecA | | 1.000 | 0.648 | 0.029* | 0.563 |  |  | -1.52 |  |
| A0A0M0EFX4 | P-2-dehydro-3-deoxyheptonate aldolase | | 1.000 | 1.000 | 0.058 | 0.011* |  |  |  | -1.76 |
| A0A0M0EG73 | Putative DEAD-box ATP-dep. RNA helicase | | 1.000 | 0.081 | 0.057 | 0.005** |  |  |  | -1.58 |
| A0A0M0EG97 | Periplasmic serine endoprotease DegP-like | | 1.000 | 1.000 | 0.049* | 0.033* |  |  | 1.52 | 2.02 |
| A0A0M0EGE6 | 3-succinoylsemialdehyde dehydrogenase | | 1.000 | 0.007** | 0.542 | 0.002** |  | 1.54 |  | -2.08 |
| A0A0M0EGJ2 | Transcriptional regulator YqjI | | 1.000 | 0.921 | 0.01* | 0.011* |  |  | 1.79 | 1.37 |
| A0A0M0EGQ7 | ATP P-ribosyltransferase regulatory sub. | | 0.659 | 0.040* | 0.258 | 1.000 |  | -1.71 |  |  |
| A0A0M0EGY8 | Riboflavin biosynthesis protein | | 0.539 | 0.630 | 0.038* | 0.009** |  |  | -1.76 | -2.07 |
| A0A0M0EH01 | Transcriptional regulatory protein CusR | | 1.000 | 1.000 | 0.074 | 0.022* |  |  |  | 2.68 |
| A0A0M0EHY7 | Uncharacterized protein | | 1.000 | 0.223 | 0.278 | 0.006** |  |  |  | 2.29 |
| A0A0M0EI08 | ABC transporter protein | | 1.000 | 0.921 | 0.001** | 0.000** |  |  | 2.72 | 4.17 |
| A0A0M0EI61 | Polymer-forming cytoskeletal | | 1.000 | 0.419 | 0.04* | 0.013* |  |  | 1.13 | 2.02 |
| A0A0M0EI81 | ATP synthase epsilon chain | | 1.000 | 0.590 | 0.037* | 0.005** |  |  |  | 1.82 |
| A0A0M0EI92 | Uncharacterized protein | | 1.000 | 1.000 | 0.011* | 0.011* |  |  | 1.73 | 1.65 |
| A0A0M0EID5 | Ribosome hibernation promoting factor (HPF) | | 1.000 | 1.000 | 0.005** | 0.003** |  |  | 1.52 | 2.08 |
| A0A0M0EIH0 | Murein hydrolase A | | 1.000 | 0.880 | 0.016* | 0.003** |  |  | 1.13 | 2.60 |
| A0A0M0EIH2 | Elongation factor G | | 0.591 | 0.765 | 0.626 | 0.009** |  |  |  | -1.49 |
| A0A0M0EIK8 | Cold shock protein CspA | | 1.000 | 1.000 | 0.004** | 0.003** |  |  | 4.64 | 3.66 |
| A0A0M0EIM6 | Uncharacterized protein | | 1.000 | 1.000 | 0.035* | 0.013* |  |  | 1.50 | 2.06 |
| A0A0M0EIP1 | Nucleoside diphosphate kinase | | 1.000 | 1.000 | 0.035* | 0.043* |  |  | 2.36 | 1.80 |
| A0A0M0EIP2 | Putative xanthine dehydrogenase YagT sub. | | 0.931 | 0.880 | 0.395 | 0.011* |  |  |  | -1.27 |
| A0A0M0EIX9 | Ribonucleoside-diphosphate reductase sub. beta | | 1.000 | 0.210 | 0.256 | 0.011* |  |  |  | -1.48 |
| A0A0M0EIY0 | Aminotransferase | | 0.752 | 0.346 | 1.000 | 0.015* |  |  |  | -1.72 |
| A0A0M0EJ26 | Cysteine synthase | | 0.565 | 1.000 | 0.423 | 0.033* |  |  |  | -1.29 |
| A0A0M0EJ72 | General stress protein 69 | | 0.744 | 1.000 | 0.024* | 0.588 |  |  | -1.70 |  |
| A0A0M0EJ94 | Glyoxalase resistance protein | | 1.000 | 1.000 | 0.016* | 0.005** |  |  | 1.57 | 2.39 |
| A0A0M0EJB9 | Outer membrane protein assembly factor BamA | | 1.000 | 1.000 | 0.032* | 0.011* |  |  | 1.73 | 2.44 |
| A0A0M0EJJ2 | Cytosol aminopeptidase | | 1.000 | 0.372 | 0.006** | 0.006** |  |  | 1.78 | 1.41 |
| A0A0M0EJM2 | Biopolymer transport protein ExbB | | 1.000 | 1.000 | 0.048* | 0.001** |  |  | 1.29 | 2.43 |
| A0A0M0EJN0 | Phospho-methylpyrimidine synthase ThiC | | 1.000 | 0.005** | 0.140 | 0.002** |  | 4.68 |  | -1.97 |
| A0A0M0EJT2 | Heat-inducible transcription repressor HrcA | | 1.000 | 1.000 | 0.004** | 0.005** |  |  | 2.46 | 1.59 |
| A0A0M0EJV2 | Paraquat-inducible protein B | | 1.000 | 1.000 | 0.163 | 0.011* |  |  |  | 2.27 |
| A0A0M0EJV7 | Ribosome maturation factor RimP | | 1.000 | 1.000 | 0.031* | 0.014* |  |  | 1.59 | 1.86 |
| A0A0M0EKA6 | All-trans-phytoene synthase | | 1.000 | 0.022* | 0.016* | 1.000 |  | -1.71 | -1.51 |  |
| A0A0M0EKB1 | Probable phosphoketolase | | 0.455 | 0.073 | 0.035* | 0.015* |  |  | -1.01 | -1.10 |
| A0A0M0EKK2 | Alcohol dehydrogenase | | 0.591 | 0.935 | 0.005** | 0.087 |  |  | -1.77 |  |
| A0A0M0EKS4 | Uncharacterized protein | | 1.000 | 1.000 | 0.013* | 0.011* |  |  | 3.19 | 2.42 |
| A0A0M0EKT8 | Translation initiation factor IF-2 | | 1.000 | 0.013* | 0.202 | 0.002** |  |  |  | -1.42 |
| A0A0M0EKU7 | Glutamyl-tRNA (Gln) amidotransferase sub. A | | 1.000 | 0.279 | 0.016* | 0.002** |  |  | -1.24 | -1.77 |
| A0A0M0EKX1 | Cytokinin riboside 5'-monophosphate P-ribohydrolase | | 1.000 | 1.000 | 0.022* | 0.007** |  |  | 1.34 | 1.79 |
| A0A0M0EKX4 | Cold shock-like protein CspE | | 1.000 | 1.000 | 0.017* | 0.007** |  |  | 1.35 | 1.61 |
| A0A0M0EKY0 | 4-hydroxy-tetrahydrodipicolinate synthase | | 1.000 | 0.206 | 0.029* | 1.000 |  |  | -2.31 |  |
| A0A0M0EL12 | Peptide chain release factor 1 | | 1.000 | 1.000 | 0.01* | 0.003** |  |  | 1.52 | 1.97 |
| A0A0M0EL53 | Adenylyl-sulfate kinase | | 0.752 | 0.481 | 0.993 | 0.014* |  |  |  | -1.86 |
| A0A0M0EL74 | Uncharacterized protein | | 0.539 | 1.000 | 0.437 | 0.007** |  |  |  | -1.85 |
| A0A0M0EL99 | Tryptophan--tRNA ligase | | 1.000 | 1.000 | 0.016* | 0.052 |  |  | -1.95 |  |
| A0A0M0ELA2 | Acetolactate synthase | | 1.000 | 1.000 | 0.016* | 0.003** |  |  | 1.27 | 2.11 |
| A0A0M0ELF1 | Glutamine--fructose-6-P aminotransferase | | 0.360 | 0.803 | 0.395 | 0.028* |  |  |  | -1.09 |
| A0A0M0ELH6 | DNA gyrase sub. B | | 1.000 | 0.015* | 0.027* | 0.000** |  |  |  | -1.47 |
| A0A0M0ELK1 | Malonyl CoA transacylase | | 0.455 | 0.013* | 0.092 | 1.000 |  | -1.75 |  |  |
| A0A0M0ELR9 | Dihydroxyacetone kinase | | 0.776 | 0.566 | 0.005** | 0.093 |  |  | -1.86 |  |
| A0A0M0ELW5 | 3-phosphoshikimate 1-carboxyvinyltransferase | | 1.000 | 0.259 | 0.035* | 0.708 |  |  | -3.50 |  |
| A0A0M0ELX6 | 50S ribosomal protein L23 | | 1.000 | 1.000 | 0.033* | 0.004** |  |  | 3.26 | 2.22 |
| A0A0M0EM17 | Outer membrane protein OprM | | 1.000 | 1.000 | 0.004** | 0.002** |  |  | 2.61 | 3.64 |
| A0A0M0EM24 | DNA-directed RNA polymerase sub. beta | | 0.752 | 0.272 | 0.726 | 0.006** |  |  |  | -1.88 |
| A0A0M0EM39 | 50S ribosomal protein L11 | | 1.000 | 1.000 | 0.243 | 0.014* |  |  |  | 2.69 |
| A0A0M0EM44 | DNA-directed RNA polymerase sub. beta' | | 1.000 | 0.079 | 0.700 | 0.011* |  |  |  | -1.82 |
| A0A0M0EM65 | 1-deoxy-D-xylulose-5-phosphate synthase | | 0.966 | 0.101 | 0.611 | 0.036* |  |  |  | -1.31 |
| A0A0M0EMB1 | Glyoxylate reductase A | | 1.000 | 0.029* | 1.000 | 0.013* |  | 1.25 |  | -1.20 |
| A0A0M0EN48 | Transcription elongation factor GreA | | 1.000 | 1.000 | 0.065 | 0.028* |  |  |  | 2.12 |
| A0A0M0EN54 | RNA polymerase sigma factor RpoD | | 1.000 | 1.000 | 0.022* | 0.014* |  |  | -2.93 | -3.06 |
| A0A0M0ENF9 | 3-isopropylmalate dehydratase large sub. | | 0.562 | 0.336 | 1.000 | 0.013* |  |  |  | -1.47 |
|  |  | |  |  |  |  |  |  |  |  |
